# Machine learning-based identification of tumor-infiltrating immune cell-associated lncRNAs for improving outcomes and immunotherapy responses in patients with low-grade glioma

**DOI:** 10.7150/thno.74281

**Published:** 2022-08-08

**Authors:** Nan Zhang, Hao Zhang, Wantao Wu, Ran Zhou, Shuyu Li, Zeyu Wang, Ziyu Dai, Liyang Zhang, Fangkun Liu, Zaoqu Liu, Jian Zhang, Peng Luo, Zhixiong Liu, Quan Cheng

**Affiliations:** 1 Department of Neurosurgery, Xiangya Hospital, Central South University, China.; 2 One-third Lab, College of Bioinformatics Science and Technology, Harbin Medical University, China.; 3 Department of Oncology, Xiangya Hospital, Central South University, China.; 4 Division of Neuroscience and Experimental Psychology, Faculty of Biology, Medicine and Health, University of Manchester, UK.; 5 Department of Thyroid and Breast Surgery, Tongji Hospital, Tongji Medical College of Huazhong University of Science and Technology, China.; 6 Department of Interventional Radiology, The First Affiliated Hospital of Zhengzhou, China.; 7 Department of Oncology, Zhujiang Hospital, Southern Medical University, China.; 8 National Clinical Research Center for Geriatric Disorders, Xiangya Hospital, Central South University, China.; 9 Department of Neurosurgery, The Second Affiliated Hospital, Chongqing Medical University, China.

**Keywords:** Immunotherapy, low-grade glioma, lncRNA, immune checkpoint, immune infiltration

## Abstract

**Rationale:** Accumulating evidence demonstrated that long noncoding RNAs (lncRNAs) involved in the regulation of the immune system and displayed a cell-type-specific pattern in immune cell subsets. Given the vital role of tumor-infiltrating lymphocytes in effective immunotherapy, we explored the tumor-infiltrating immune cell-associated lncRNA (TIIClncRNA) in low-grade glioma (LGG), which has never been uncovered yet.

**Methods:** This study utilized a novel computational framework and 10 machine learning algorithms (101 combinations) to screen out TIIClncRNAs by integratively analyzing the sequencing data of purified immune cells, LGG cell lines, and bulk LGG tissues.

**Results:** The established TIIClnc signature based on the 16 most potent TIIClncRNAs could predict outcomes in public datasets and the Xiangya in-house dataset with decent efficiency and showed better performance when compared with 95 published signatures. The TIIClnc signature was strongly correlated to immune characteristics, including microsatellite instability, tumor mutation burden, and interferon γ, and exhibited a more active immunologic process. Furthermore, the TIIClnc signature predicted superior immunotherapy response in multiple datasets across cancer types. Notably, the positive correlation between the TIIClnc signature and CD8, PD-1, and PD-L1 was verified in the Xiangya in-house dataset.

**Conclusions:** The TIIClnc signature enabled a more precise selection of the LGG population who were potential beneficiaries of immunotherapy.

## Introduction

Low-grade glioma (LGG) is a heterogeneous group of neuroepithelial tumors derived from supporting glial cells 1 and includes grades I and II out of four glioma grades classified by the World Health Organization (WHO) 2. Given the superior malignancy of glioblastoma (grade IV glioma), The Cancer Genome Atlas (TCGA) database classifies grades II and III gliomas as LGG. The outcomes of LGG are highly variable, depending on age at diagnosis, histological subtype, tumor size, etc. 3. Surgical resection with postoperative radiotherapy and chemotherapy is the conventional treatment for LGG patients 4. Despite its relatively benign biological characteristics, LGG remains incurable and slowly develops till premature death 1. Numerous recent studies have focused on the tumor immune microenvironment (TIME) and the interactions between tumor and immune cells 5. Immunotherapy based on the immune response, including immune checkpoint blockade (ICB) and adoptive cell transfer (ACT), has revolutionized the therapeutic outcomes for tumor patients. Researchers have made unremitting efforts to find more precise targets for the further development of immunotherapy. However, studies on immunotherapy of LGG remain to be enriched.

Long noncoding RNAs (lncRNAs), a group of noncoding RNAs with more than 200 nucleotides, are closely related to diversified biological functions 6. Many studies demonstrated that lncRNAs played a critical role in regulating cellular biological processes through modulating gene expression at transcriptional, post-transcriptional, and epigenetic levels 7. Furthermore, the latest research has found that lncRNAs participated in the immune system modulation and showed a cell-type-specific pattern in immune cell subsets 8. Ranzan et al. investigated over 500 lncRNAs and identified T cell-specific lncRNAs regulating cell differentiation 9. Another study found lncRNA SATB2-AS1 as an essential regulator in colorectal cancer progression and immune cell density 10. Given their close association with immune cell infiltration 8, 10, lncRNAs have colossal potential to evaluate immunotherapy response and predict clinical outcomes. Thus, it is promising to incorporate these powerful lncRNAs to develop prognostic biomarkers based on bioinformatics technology. Besides, as scientific research has entered the big data era with the fast development of high-throughput sequencing technologies, machine learning has been gradually widely applied to extract essential knowledge from big data bioinformatics.

This study aimed to screen tumor-infiltrating immune cell-associated lncRNA (TIIClnc) through a novel computational framework. Via integrative analysis of sequencing data of purified immune cells, LGG cell lines, bulk LGG tissues, and machine learning algorithms, we established a TIIClnc signature to stratify LGG patients and predict the outcomes of immunotherapy.

## Materials and methods

### LGG patient and tumor cell line cohorts collection

Transcriptome data and clinical information of LGG patients were accessed from three databases, The Cancer Genome Atlas (TCGA, https://portal.gdc.cancer.gov) via Illumina-HiSeq platform, Chinese Glioma Genome Atlas (CGGA, http://www.cgga.org.cn/) via Illumina-HiSeq platform, and Gene Expression Omnibus (GEO, http://www.ncbi.nlm.nih.gov/geo) via Affymetrix Human Genome U133 Plus 2.0 Array platform. The total number of samples included in this study was 932, including 518 samples from the TCGA LGG dataset, 77 samples from the Xiangya in-house dataset, 169 samples from the CGGA LGG dataset, and 168 samples from the GSE108474 dataset. The immune-associated lncRNA signature was constructed through the TCGA LGG training dataset and subsequently validated using the Xiangya in-house dataset (Glioma tissues were collected and written informed consent was obtained from all patients. The included glioma tissues were approved by the Ethics Committee of Xiangya Hospital, Central South University). CGGA LGG and GSE108474 datasets were used as the external validating datasets. Transcriptome data of 10 LGG cell lines via the Affymetrix Human Genome U133 Plus 2.0 Array platform was accessed from GSE36133 (Cancer Cell Line Encyclopedia project (CCLE).

### Purified immune cell line cohorts collection

Transcriptome data of 115 purified cell lines from 19 major immune cell types via Affymetrix Human Genome U133 Plus 2.0 Array platform was accessed from 16 datasets, including GSE27291, GSE27838, GSE28490, GSE13906, GSE23371, GSE25320, GSE28698, GSE28726, GSE49910, GSE51540, GSE59237, GSE37750, GSE39889, GSE42058, GSE6863, GSE8059, and processed as previously described 8.

### Preprocessing of transcriptome data

The Robust Multi-array Average (RMA) algorithm 11 from the R package affy was used to perform quantile normalization, background correction, and log2 transformation of microarray data from the Affymetrix platform. The sequencing data fragments per kilobase million (FPKM) values were changed into transcripts per kilobase million (TPM) values. The probes of microarray data from the Affymetrix platform were renamed to obtain lncRNA expression profiles. After matching the annotation file of GENCODE (release 39) with the NetAffx annotation files (release 36), probe sets with Ensembl gene IDs as 'long non-coding RNA' was picked out. 1711 characteristic lncRNAs matching with 2019 probes in microarray data from the Affymetrix platform were selected for further analysis. Correspondingly, lncRNA expression profiles (IlluminaHiSeq platform) were accessed from the TCGA and CGGA databases.

### Tumor-infiltrating immune cell-associated lncRNA signature establishment

**Figure 1:**
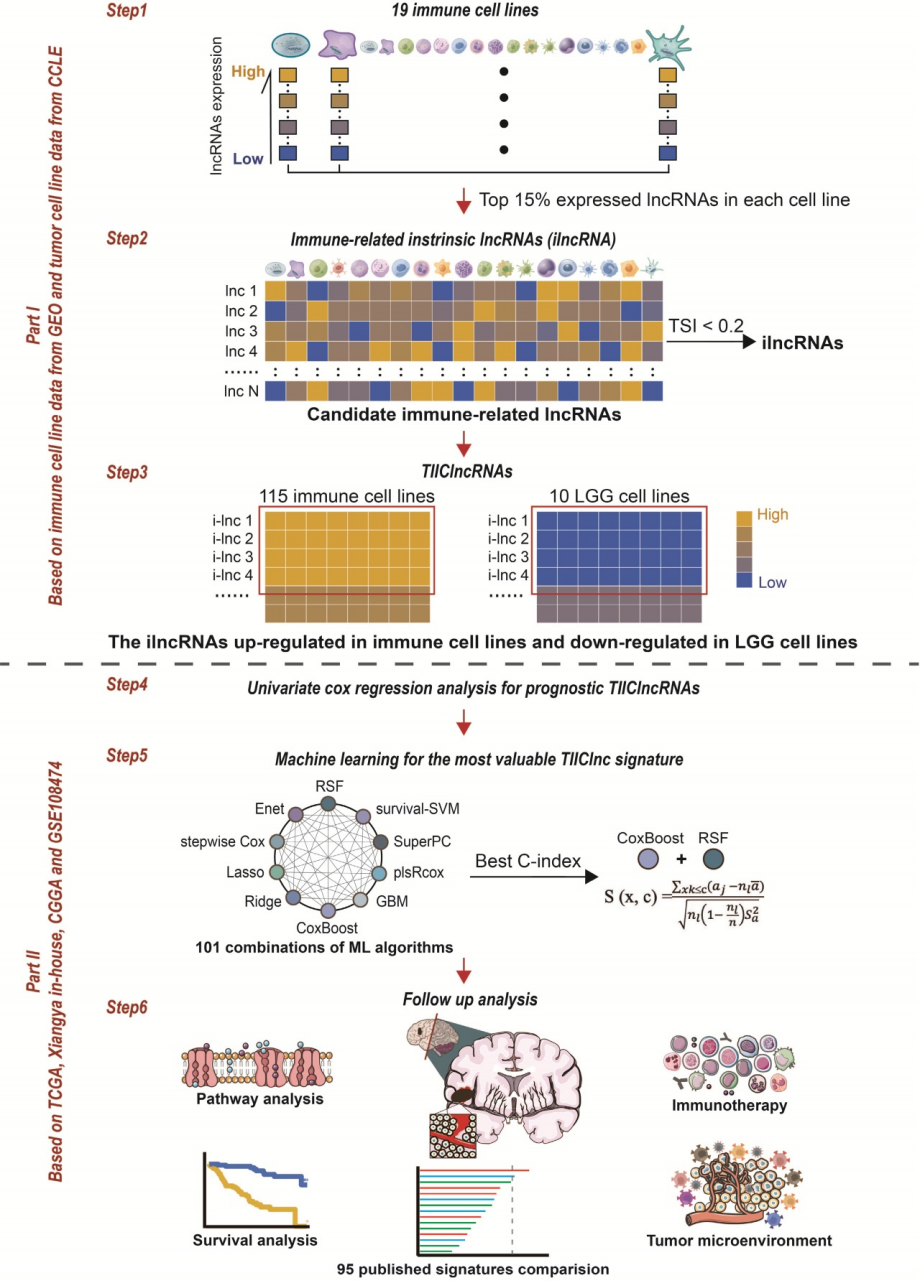
** The computational framework for establishing the TIIClnc signature.** The top 15% expressed lncRNAs were taken for candidate immune-related lncRNAs for each immune cell line. TSI was applied to calculate the expression specificity of candidate immune-related lncRNAs for each cell type. The highly expressed lncRNAs in all immune cell types were identified as immune-related ilncRNA. ilncRNAs significantly upregulated in immune cell lines and downregulated in LGG cell lines were defined as TIIClncRNAs. Univariate cox regression analysis was further used to screen out the prognostic TIIClncRNAs. All combinations of 10 machine learning algorithms, including RSF, Enet, Lasso, Ridge, stepwise Cox, CoxBoost, plsRcox, SuperPC, GBM, and survival-SVM, based on a 10-fold cross-validation were further used to screen out the most valuable TIIClnc signature with the highest C-index. The TIIClnc signature was finally generated based on the combination of RSF and CoxBoost. The association between the TIIClnc signature, prognosis, tumor immune microenvironment, and immunotherapy response was comprehensively investigated.

Through integrative lncRNA profiling analysis of purified immune cells, LGG cell lines, and bulk LGG cancer tissues, a novel computational framework was utilized to identify a tumor-infiltrating immune cell-associated lncRNA (TIIClnc) signature on account of a couple of machine learning algorithms. The details are as follows (Figure 1):

- The top 15% expressed lncRNAs (each immune cell line) were taken for candidate immune-related lncRNAs.
- The tissue specificity index (TSI) proposed by Yanai et al. 12 was applied to calculate the expression specificity of candidate lncRNAs for each cell type:

TSIlnc = 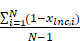


where N represents the total number of immune cell types and x_lnc, i_ represents the expression level of immune cell i normalized by the maximal expression intensity of lncRNA in any immune cell type. TSI ranges from 0 to 1, in which lncRNA is defined as an immune cell-general lncRNA when the value is 0 or an immune cell-specific lncRNA when the value is 1. The highly expressed lncRNAs in all immune cell types were identified as immune-related intrinsic lncRNAs (ilncRNA).

- ilncRNAs differentially expressed between immune cell lines (upregulated) and LGG cell lines (downregulated) were determined as TIIClncRNAs.
- Univariate Cox regression analysis was subsequently used to filtrate the TIIClncRNAs with prognostic potential in the TCGA LGG dataset.
- 101 combinations of 10 machine learning algorithms, including Lasso, Ridge, stepwise Cox, CoxBoost, random survival forest (RSF), elastic network (Enet), partial least squares regression for Cox (plsRcox), supervised principal components (SuperPC), generalized boosted regression modeling (GBM), and survival support vector machine (survival-SVM) based on a 10-fold cross-validation were further used to screen out the most valuable TIIClnc signature with the highest C-index.
- The TIIClnc signature was established based on the combination of RSF and CoxBoost. CoxBoost algorithm was used to screen out the most valuable TIIClncRNAs. RSF algorithm was further used to filtrate the most reliable model. Log-rank score test for splitting survival trees was conducted as previously described 13. First, the x-variable x was assumed to be ordered as x_1_ ≤ x_2_ ≤ … ≤ x_n_. Then, the “ranks” for each survival time T_j_ (j ∈ [1, …, n]) were computed. The obtained equation is as follows:

a_j_ = δ_j_ - 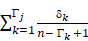


where 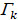 = #[t : T_t_ ≤ T_k_] and 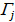 represents the index of the order for T_j_. The log-rank score test came as follows:

TIIClnc signature = S (x, c) =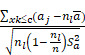


where 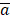 and 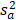 represent the sample mean and sample variance of [a_j_ : j = 1, . . . , n], respectively. The measure of node separation is determined using log-rank score splitting by | S (x, c) |. The best split is reached by maximizing this value over x and c.

### Annotation of immune-related characteristics for the TIIClnc signature

Seven types of immune modulators were collected 14. T cell-inflamed gene expression profile (GEP), Cytotoxic activity (CYT), and interferon γ (IFN-γ) were calculated 15, 16. Tumor mutation burden (TMB), microsatellite instability (MSI), T cell receptor (TCR) richness, TCR Shannon, and SNV Neoantigen were collected from the TCGA database. GATK4 was used to search for the SNPs and indels from the RNA sequencing data of the Xiangya in-house dataset. ANNOVAR was used to annotate the mutation information based on CRCh38 17. The tmb function of the R package maftools was applied further to calculate the TMB value of the Xiangya in-house dataset. The R package PreMSIm was used to predict the MSI value of the Xiangya in-house dataset. Six immune subtypes and immunophenoscore (IPS) were determined as previously described 14, 18. The Tumor Immune Estimation Resource (TIMER) algorithm 19, single cell gene set enrichment analysis (ssGSEA) algorithm 18, Microenvironment Cell Populations-counter (MCPcounter) algorithm 20, and Estimation of STromal and Immune cells in MAlignant Tumours using Expression data (ESTIMATE) algorithm 21 were applied for calculating the abundance of immune infiltrating cells and ESTIMATE score. Cancer immunity cycle, displaying the functional status of chemokines and immunomodulators, and 114 metabolic pathways were collected and calculated using gene set variation analysis (GSVA) 22-24. TIME signatures independently developed by Kobayashi 25 and Bagaev 26 were collected and calculated using GSVA. Gene Ontology (GO) and Kyoto Encyclopedia of Genes and Genomes (KEGG) terms were also quantified using GSVA and gene set enrichment analysis (GSEA).

### Predictive value of the TIIClnc signature for immunotherapy response

The GSE35640 (melanoma) 27, GSE91061 (melanoma) 28, GSE78220 (melanoma) 29, Allen (melanoma) 30, Nathanson (melanoma) 31, IMvigor (urothelial carcinoma) 32, Braun (renal cell carcinoma) 33, GSE179351 (colorectal adenocarcinoma and pancreatic adenocarcinoma) 34, GSE165252 (esophageal adenocarcinoma) 35, and PRJNA482620 (glioblastoma) 36 datasets were used to predict the immunotherapy response, while the TIIClnc signature was calculated in each dataset. The GSE103668 (triple-negative breast cancer) 37 dataset was used to predict the targeted therapy response (cisplatin & bevacizumab). The subclass mapping was utilized to predict anti-PD-1 and anti-CTLA-4 immunotherapy responses 38. The Tumor Immune Dysfunction and Exclusion (TIDE) algorithm was also used in this section 39.

### RNA sequencing

RNAstore-fixed tumor tissues of 77 LGG samples from the Xiangya in-house dataset (Table S1) were collected for RNA sequencing as previously described 40. Briefly, total RNA was extracted from the tumor tissues using TRIzol (Sigma-Aldrich, CA, USA). RNA purity and RNA integrity were checked using the NanoPhotometer spectrophotometer (IMPLEN, CA, USA) and the RNA Nano 6000 Assay Kit of the Bioanalyzer 2100 system (Agilent Technologies, CA, USA), respectively. A total amount of 1 μg RNA per sample was used as input material for the RNA sample preparations. Sequencing libraries were generated using NEBNext® UltraTM RNA Library Prep Kit for Illumina® (NEB, USA).

### Immunohistochemistry staining

Paraffin-embedded tumor tissues of 20 of 77 LGG samples used for RNA sequencing from the Xiangya in-house dataset were further collected for immunohistochemistry (IHC) staining. Briefly, tissue sections were placed in citric acid antigen repair buffer (pH 6.0) in a microwave oven for antigen retrieval. Slices were placed in a 3% hydrogen peroxide solution to block endogenous peroxidase. 3% bovine serum albumin (BSA) was used as a blocking reagent. The sections were incubated with primary antibodies against CD8 (Mouse, 1:1000, 66868-1-Ig, Proteintech, China), PD-1 (Rabbit, 1:800, 18106-1-AP, Proteintech, China), PD-L1 (Mouse, 1:1000, 66248-1-Ig, Proteintech, China). The sections were then incubated with a horseradish peroxidase-conjugated secondary antibody (1:200, GB23303, Servicebio, China). 3-3'-diaminobenzidine (G1211, Servicebio, China) was finally used for coloration, and hematoxylin was used for counterstaining cell nuclei.

### RT-qPCR assay

The primers of GAPDH (F ACAGCCTCAAGATCATCAGC; R GGTCATGAGTCCTTCCACGAT), LOC101928134 (F GAGCGAGGGTGATTGTCCA; R GAAGAGGGGAAGGGGTTCTC), and LOC100133461 (F GAGAGACCTGCCCAAGCATT; R TCCAGGTTCTGCATGTGTCC) were designed using the primer 5.0. Total RNAs were extracted and reversely transcribed into cDNA by HiScript Q RT SuperMix for RT-qPCR. The expression levels of LOC101928134 and LOC100133461 were quantified using 2-ΔΔCT.

### Cell Counting Kit-8 (CCK-8) assay

The THP-1 cells were cultured in 1640 medium with 10% fetal bovine serum (FBS). The THP-1 cells were seeded in a 96-well plate at 10^4^ cells/hole density. Three groups of the THP-1 cells transfected with siRNAs were cultured for 24 h, 48 h, and 72 h, respectively. Each group has four duplicated holes. The absorbance at 450 nm was measured after hatching under the condition of 37 ℃ and 5% CO2.

### EdU assay

The EdU (5-ethynyl-2'-deoxyuridine) assay was performed according to the manufacturer's instructions (BeyoClick™ EdU Cell Proliferation Kit with Alexa Fluor 488, China). Three groups of the THP-1 cells transfected with siRNAs were incubated overnight with 50 μL 50 μM EdU medium and then fixed with 50 μL 4% paraformaldehyde. 100 μL 1x Apollo reaction solution was added for incubation for 30 min. Finally, the cells were incubated with 1 mL 1 × Hoechst 33342 reaction solution for 10 min and observed with a confocal microscope.

### Transwell assay

Three groups of the THP-1 cells transfected with siRNAs were centrifuged and resuspended using the serum-free medium. The density was adjusted to 10^5^ cells/mL. 100 μL cell suspension was added to the upper chamber, and 500 μL 1640 with 10% FBS was added to the lower chamber. After culturing for 48 h, the migrated THP-1 cells in the lower chamber were collected and counted by flow cytometry.

### Statistical analysis

Differentially expressed lncRNAs between immune and LGG cell lines were extracted by R packages 'limma'. Samples were grouped based on the cutoff value of the TIIClnc signature determined by the R package 'survminer'. The Kaplan-Meier survival plots were applied to estimate overall survival (OS) between the two TIIClnc signature groups using the R package 'survival.' C-index of OS was performed on the individual clinical variables, including the TIIClnc signature. The calibration curves of the TIIClnc signature were generated using the R package 'pec'. The predictive value of the TIIClnc signature for prognosis was measured with time-dependent receiver operating characteristic (ROC) curves using the R package 'timeROC.' For normally distributed variables, statistical differences between groups were determined by a two‐tailed t-test while a one‐way ANOVA test determined statistical differences among groups. For nonnormally distributed variables, statistic differences between groups were determined by a Wilcoxon test while a Kruskal-Wallis test determined statistic differences among groups. All the statistical analyses were performed in the R project, version 4.1.2.

## Results

### Identification of TIIClncRNAs

To comprehensively evaluate the immune cell-related lncRNA, 115 purified cell lines from 19 major immune cell types from 16 datasets were collected by searching for literature from 2007 to 2022 (Figure 1). 546 lncRNAs (top 15% expressed lncRNAs) were taken for candidate immune-related lncRNAs selection in each immune cell line. TSI score of the 546 lncRNAs was calculated to identify the ilncRNAs generally expressed in all 19 immune cell types (Table S2). Note that lncRNAs with a lower TSI score are generally highly expressed in different immune cell types, indicating their fundamental roles in immunity. 308 ilncRNAs were confirmed to be critical for regulating elemental immunity with the threshold of TSI < 0.2. By comparing the expression of 308 ilncRNAs in immune cell lines and LGG cell lines, 136 ilncRNAs were further found to be significantly upregulated in 115 immune cell lines and downregulated in 10 LGG cell lines. These 136 ilncRNAs were defined as LGG TIIClncRNAs.

### Development of the TIIClnc signature

**Figure 2:**
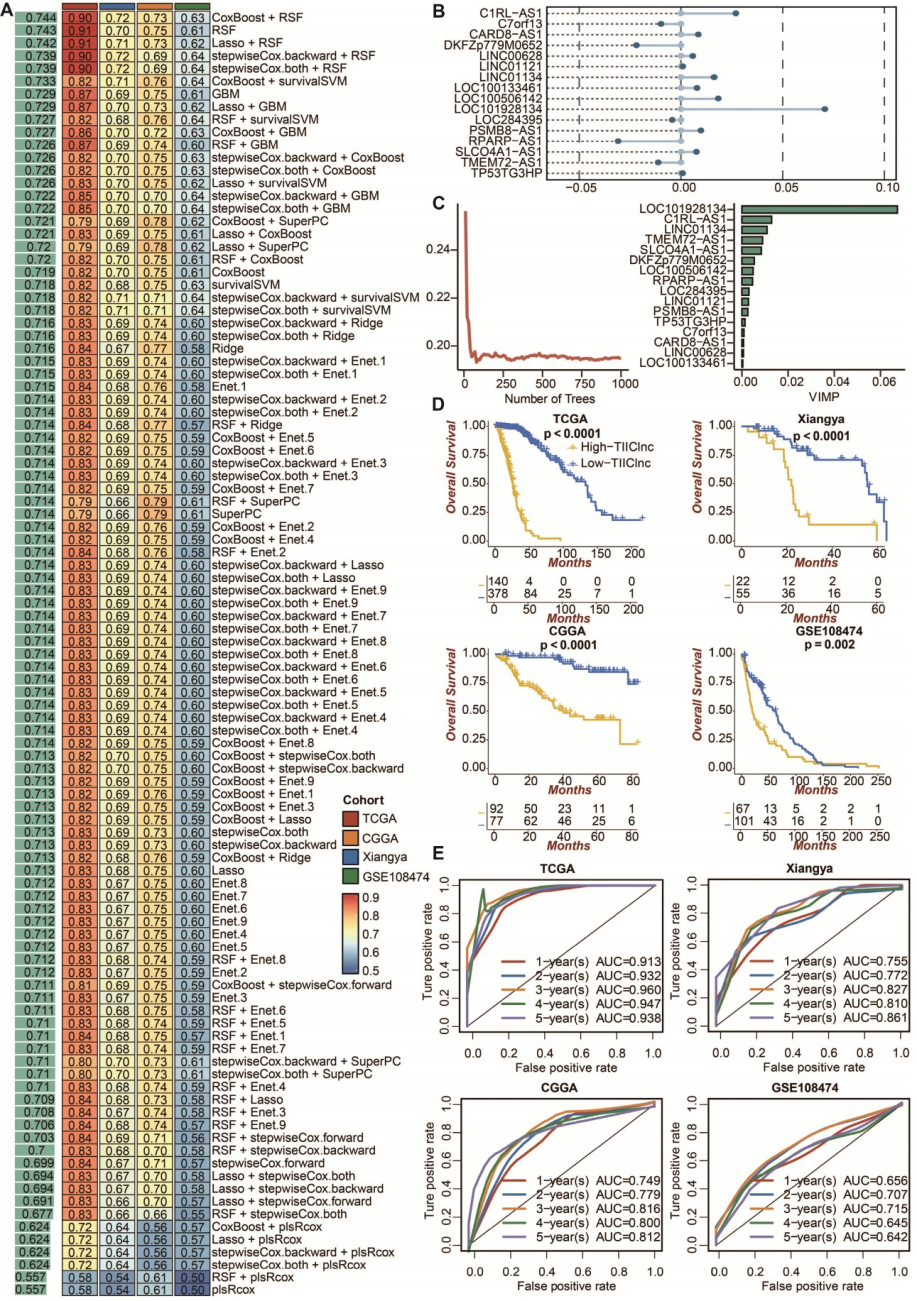
** The prognostic value of the TIIClnc signature. A.** A total of 101 combinations of machine learning algorithms for the TIIClnc signatures via a 10-fold cross-validation framework. The C-index of each model was calculated across validation datasets, including TCGA LGG, Xiangya in-house, CGGA LGG, and GSE108474 datasets. **B.** The exhibition of the 16 most valuable TIIClncRNAs based on the CoxBoost algorithm. **C.** The number of trees for determining the TIIClnc signature with minimal error and the importance of the 16 most valuable TIIClncRNAs based on the RSF algorithm. **D.** Kaplan-Meier survival curve of OS between patients with a high score of TIIClnc signature and with a low score of TIIClnc signature in the TCGA LGG, Xiangya in-house, CGGA LGG, and GSE108474 datasets. **E.** Time-dependent ROC curves of 1-year, 2-year, 3-year, 4-year, and 5-year OS in the CGGA LGG, Xiangya in-house, TCGA LGG, and GSE108474 datasets.

To develop a prognostic TIIClnc signature, univariate Cox regression analysis regarding OS confirmed 46 prognostic TIIClncRNAs from 136 ilncRNAs in the TCGA LGG dataset. 10 machine learning algorithms, including RSF, Enet, stepwise Cox, CoxBoost, plsRcox, Lasso, Ridge, SuperPC, GBM, and survival-SVM, were combined based on a 10-fold cross-validation to identify the most robust TIIClnc signature with the highest C-index in the TCGA LGG training dataset, Xiangya in-house validating dataset, and two external validating datasets (CGGA LGG and GSE108474) (Figure 2A). A final TIIClnc signature with the best performance was established based on the combined RSF and CoxBoost algorithms, which CoxBoost algorithm identified the 16 most valuable TIIClncRNAs (C7orf13, LINC00628, LINC01121, LOC100133461, LINC01134, TP53TG3HP, SLCO4A1-AS1, C1RL-AS1, LOC284395, TMEM72-AS1, PSMB8-AS1, DKFZp779M0652, LOC100506142, RPARP-AS1, CARD8-AS1, LOC101928134) (Figure 2B, Table S3) and RSF algorithm filtrated the most reliable model (Figure 2C). In accordance with our previous analyses, 16 TIIClncRNAs were generally expressed in all 19 immune cell types (Figure S1A). Besides, 16 TIIClncRNAs were significantly differentially expressed in immune cell lines (upregulation) and LGG cell lines (downregulation) (Figure S1B). Notably, LGG patients with high expression of 5 TIIClncRNAs had increased survival time, while LGG patients with high expression of 11 TIIClncRNAs had decreased survival time in the TCGA LGG dataset (Figure S2).

### *In vitro* validation of TIIClncRNAs

Among the 16 TIIClncRNAs, LOC100133461 and LOC101928134, with relatively higher expression in immune cell lines, were selected to further validate their potential roles in the TIME. RNA expression of LOC100133461 and LOC101928134 was significantly inhibited in three siRNAs groups of THP-1 cells, respectively (Figure S3A). The CCK-8 assay revealed the increased proliferation ability of THP-1 cells with inhibited expression of LOC100133461 and LOC101928134, respectively (Figure S3B and S3C). The EdU assay further proved the significantly increased proliferation ability of THP-1 cells with inhibited expression of LOC100133461 and LOC101928134, respectively (Figure S3D, S3E, S3F, S3G). Moreover, the Transwell assay revealed the significantly increased migration ability of THP-1 cells with inhibited expression of LOC100133461 and LOC101928134, respectively (Figure S3H, S3I, S3J, S3K).

### Prognostic value of the TIIClnc signature

**Figure 3:**
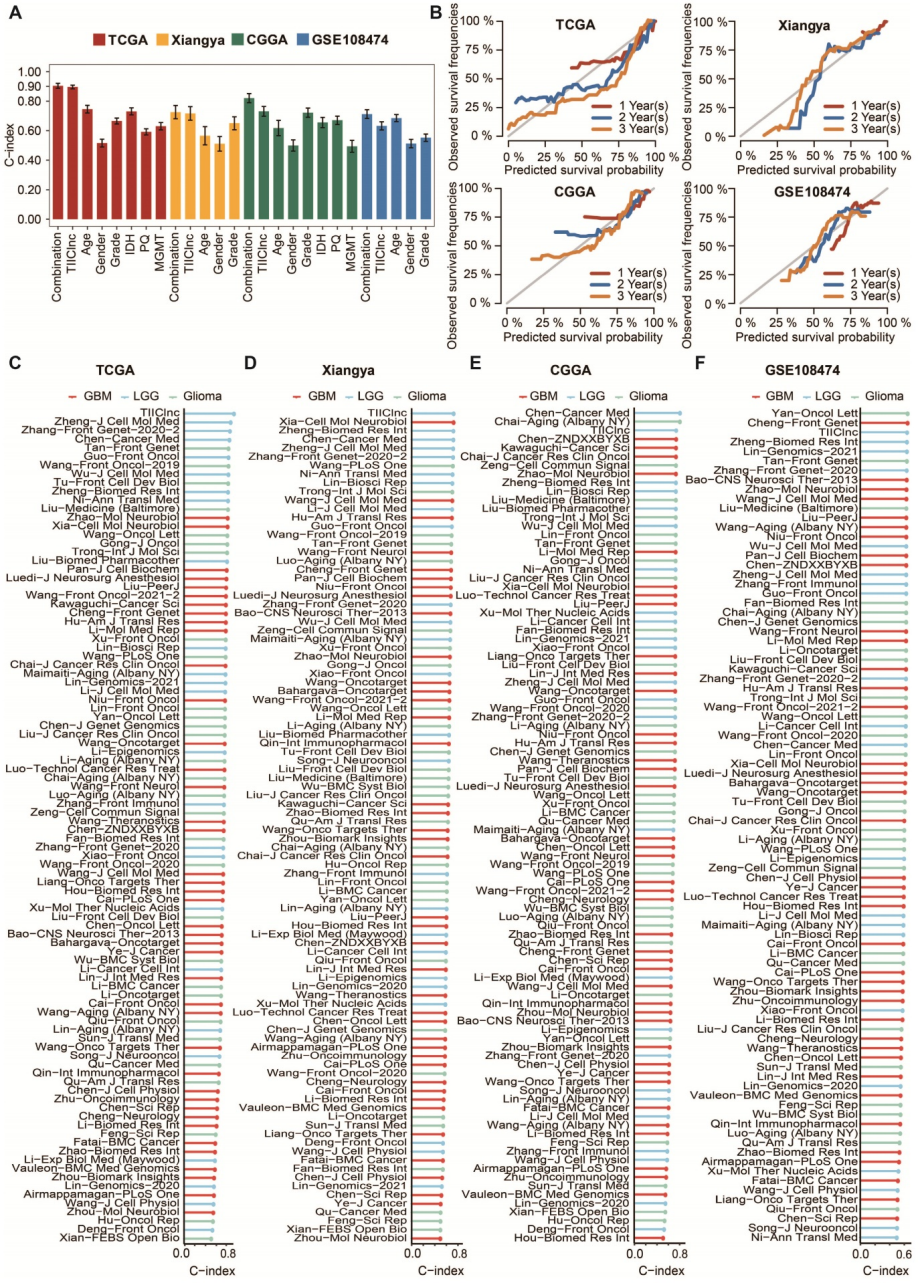
** Comparison between the TIIClnc signature and other models. A.** The C-index of the TIIClnc signature, other clinical factors, and the combination signature in the TCGA LGG, Xiangya in-house, CGGA LGG, and GSE108474 datasets. **B.** The 1-year, 2-year, and 3-year calibration curves of the TIIClnc signature in the TCGA LGG, Xiangya in-house, CGGA LGG, and GSE108474 datasets. **C.** The C-index of the TIIClnc signature and other models developed in the TCGA LGG dataset. **D.** The C-index of the TIIClnc signature and other models developed in the Xiangya in-house dataset. **E.** The C-index of the TIIClnc signature and other models developed in the CGGA LGG dataset. **F.** The C-index of the TIIClnc signature and other models developed in the GSE108474 dataset.

LGG patients with high TIIClnc signature scores had decreased survival time in the TCGA LGG, Xiangya in-house, CGGA LGG, and GSE108474 datasets (Figure 2D). Consistently, time-dependent ROC curves of 1-year, 2-year, 3-year, 4-year, and 5-year OS in the TCGA LGG dataset (AUC values, 0.913, 0.932, 0.960, 0.947, 0.938, respectively), Xiangya in-house dataset (AUC values, 0.755, 0.772, 0.827, 0.810, 0.861, respectively), CGGA LGG dataset (AUC values, 0.749, 0.779, 0.816, 0.800, 0.812, respectively), and GSE108474 dataset (AUC values, 0.656, 0.707, 0.715, 0.645, 0.642, respectively) confirmed the prognostic value of the TIIClnc signature (Figure 2E). The C-index of clinical factors in LGG patients was calculated. The univariate and multivariate Cox regression analysis was performed on clinical factors. The TIIClnc signature was found to be an independent prognostic factor like age, gender, grade, isocitrate dehydrogenase (IDH) status, 1p/19q status, methylation of O6-methylguanine-DNA methyltransferase (MGMT) status, with an advantage over all these clinical factors (Figure 3A, Table S4). Notably, the combination signature incorporating the TIIClnc signature, age, gender, grade, IDH status, 1p/19q status, and MGMT status showed better predictive efficacy in prognosis (Figure 3A). The calibration curves further proved the predictive accuracy of the TIIClnc signature (Figure 3B).

### Comparison of prognostic signatures in gliomas

Thanks to the development of next-generation sequencing and technologies for mining big data, predictive gene expression-based signatures have been widely explored and developed. For a comprehensive comparison of the performance of the TIIClnc signature with other signatures, the published signatures over the past ten years were systematically retrieved. Ultimately, 95 signatures (including mRNA and lncRNA signatures) were enrolled in this study (Table S5). These 95 signatures were closely related to different biological features, including immunotherapy response, immune infiltration, autophagy, ferroptosis, pyroptosis, stemness, epithelial-mesenchymal transition, hypoxia, glycolysis, epigenetics, N6-methyladenosine, and aging. Notably, the TIIClnc signature displayed better performance regarding C-index in the TCGA LGG (Figure 3C), Xiangya in-house (Figure 3D), CGGA LGG (Figure 3E), and GSE108474 (Figure 3F) datasets than almost all models.

### Immune characteristics related to the TIIClnc signature

**Figure 4:**
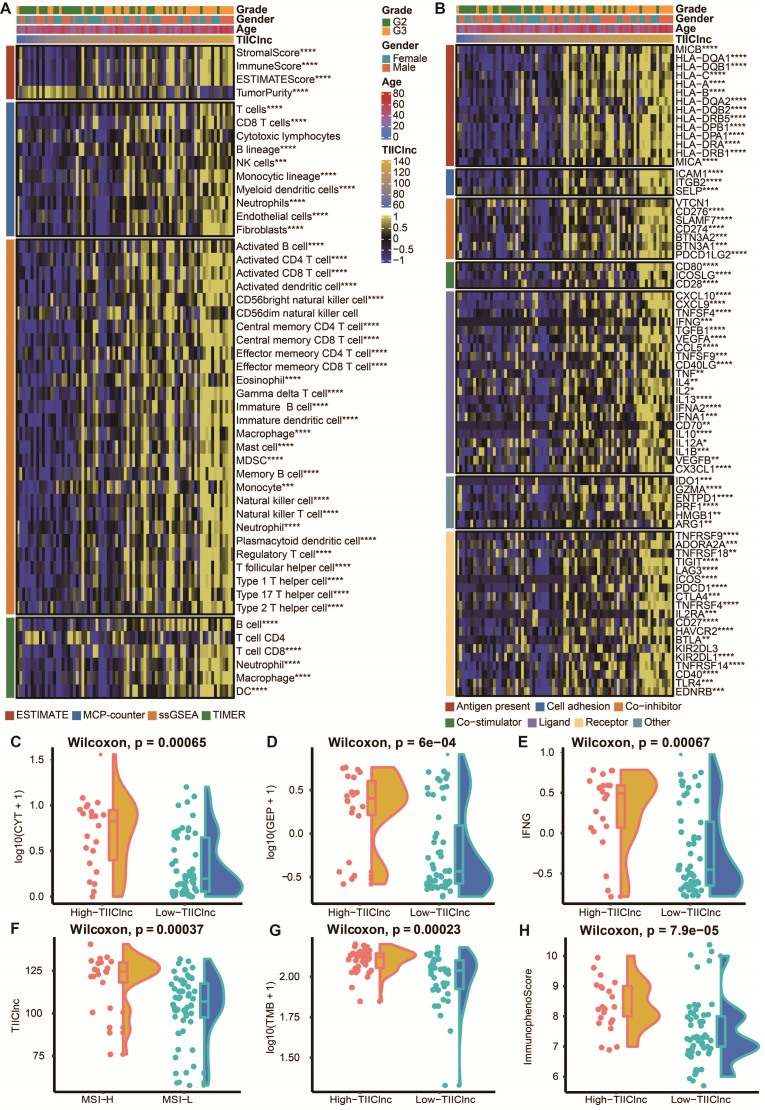
** Immune-related characteristics of the TIIClnc signature in the Xiangya in-house dataset. A.** Heatmap displaying the correlation between the TIIClnc signature and immune infiltrating cells. **B.** Heatmap displaying the correlation between the TIIClnc signature and immune modulator molecules. **C.** Box plot displaying the CYT levels between two TIIClnc signature score groups. **D.** Box plot displaying the GEP levels between two TIIClnc signature score groups. **E.** Box plot displaying the IFN-γ levels between two TIIClnc signature score groups. **F.** Box plot displaying the TIIClnc levels between two MSI groups. **G.** Box plot displaying the TMB levels between two TIIClnc signature score groups. **H.** Box plot displaying the IPS levels between two TIIClnc signature score groups.

To assess the role of the TIIClnc signature in the TIME of LGG, the relationship between the TIIClnc signature and immune cell infiltration and immune modulators was explored. Based on the TIMER algorithm, MCPcounter algorithm, and ssGSEA algorithm, the TIIClnc signature score was found to be positively correlated with almost all tumor immune infiltrating cells, such as T cells, T helper (Th) cells, natural killer (NK) cells, B cells, dendritic cell (DC), mast cells, myeloid-derived suppressor cell (MDSC), fibroblasts, macrophages, and regulatory T (Treg) cells in the TCGA LGG (Figure S4A), Xiangya in-house (Figure 4A), CGGA LGG (Figure S6A), and GSE108373 (Figure S7A) datasets. Based on the ESTIMATE algorithm, the TIIClnc signature score was positively correlated with a stromal score, immune score, and ESTIMATE score except for tumor purity in the TCGA LGG (Figure S4A), Xiangya in-house (Figure 4A), CGGA LGG (Figure S6A), and GSE108373 (Figure S7A) datasets. In addition, the TIIClnc signature score positively correlated with most immune modulators categorized into antigen presentation, cell adhesion, co-inhibitor, co-stimulator, ligand, receptor, and others in the TCGA LGG (Figure S4B), Xiangya in-house (Figure 4B), CGGA LGG (Figure S6B), and GSE108373 (Figure S7B) datasets. Notably, a strong correlation was observed between the TIIClnc signature score and classical immune checkpoint molecules, including IDO-1, LAG-3, PD-1, PD-L1, PD-L1, CTLA-4, BTLA, and TIGIT (Figure 4B, S4B, S6B, S7B).

### The TIIClnc signature is a predictive biomarker for immunotherapy response

To further explore the role of the TIIClnc signature in immunotherapy response, the relationship between the TIIClnc signature and several immunotherapy predictors was explored (Table S6). Notably, the MSI (Figure S5A), TMB (Figure S5B), CYT (Figure S5C), GEP (Figure S5D), TCR richness (Figure S5E), TCR Shannon (Figure S5F), SNV Neoantigen (Figure S5G), and IFN-γ (Figure S5H) levels were all significantly higher in the high TIIClnc signature score group in the TCGA LGG dataset, all of which were determinants of better immunotherapy response. The significant expression differences of CYT (Figure 4C, S6C, S7C), GEP (Figure 4D, S6D, S7D), and IFN-γ (Figure 4E, S6E, S7E) between two TIIClnc signature score groups were also verified in the Xiangya in-house, CGGA LGG, and GSE108474 datasets. Furthermore, the MSI high group was significantly associated with high TIIClnc signature scores (Figure 4F), and the TMB had a substantially higher level in the high TIIClnc signature score group (Figure 4G). Besides, a high TIIClnc signature score was more associated with lymphocyte depleted and inflammatory immune subtypes and high IPS score in the TCGA LGG dataset (Figure S5I), which the significant expression difference of IPS score between the two TIIClnc signature score groups was also verified in the Xiangya in-house, CGGA LGG, and GSE108474 datasets (Figure 4H, S6F, S7F).

**Figure 5:**
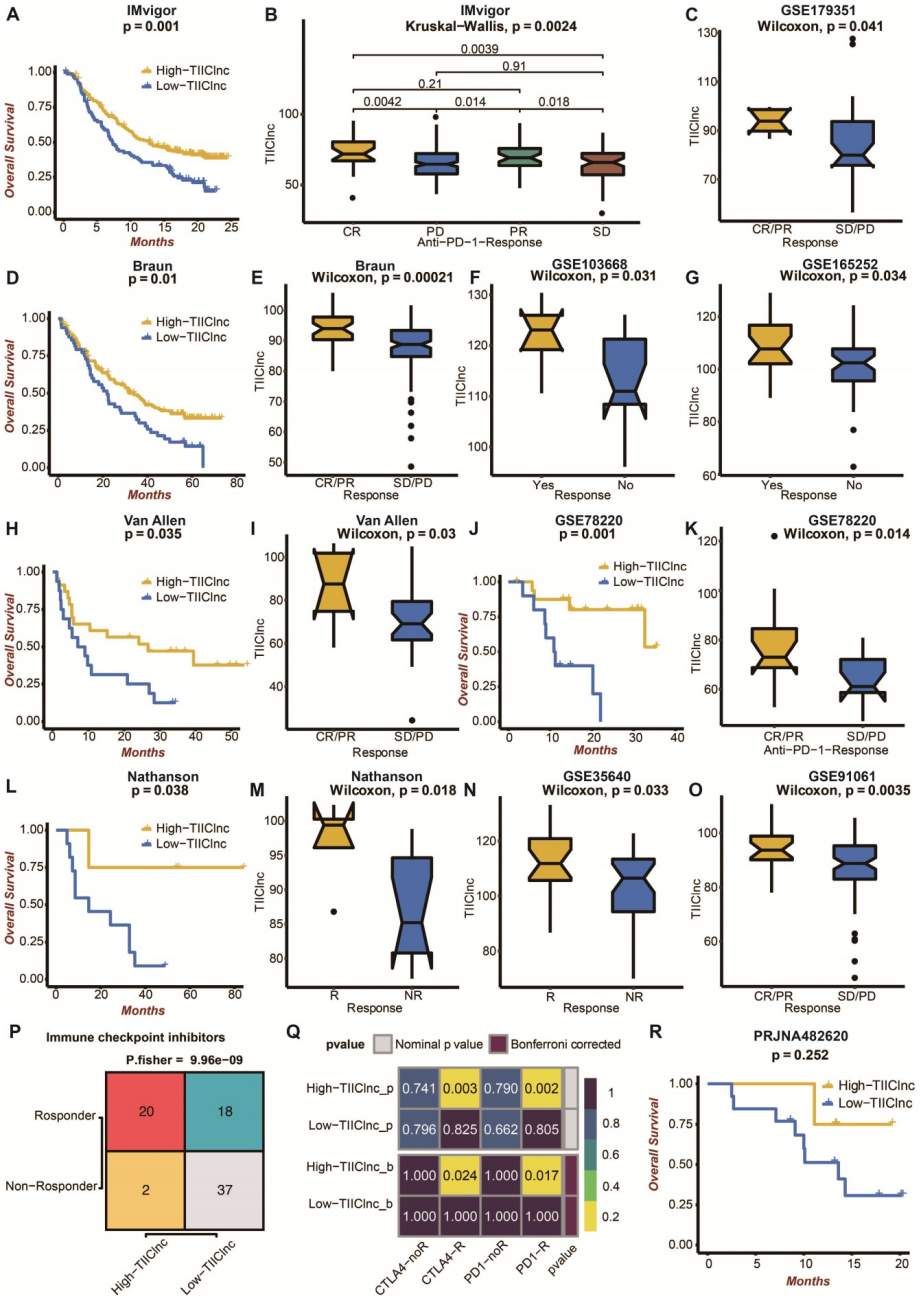
** Predictive value of the TIIClnc signature in immunotherapy response. A.** Kaplan-Meier survival curve of OS between patients with a high score of TIIClnc signature and a low score of TIIClnc signature in the IMvigor dataset. **B.** Box plot displaying the TIIClnc signature score in patients with different immunotherapy responses in the IMvigor dataset. **C.** Box plot displaying the TIIClnc signature score in patients with different immunotherapy responses in the GSE179351 dataset. **D.** Kaplan-Meier survival curve of OS between patients with a high score of TIIClnc signature and a low score of TIIClnc signature in the Braun dataset. **E.** Box plot displaying the TIIClnc signature score in patients with different immunotherapy responses in the Braun dataset. **F.** Box plot displaying the TIIClnc signature score in patients with different immunotherapy responses in the GSE103668 dataset. **G.** Box plot displaying the TIIClnc signature score in patients with different immunotherapy responses in the GSE165252 dataset. **H.** Kaplan-Meier survival curve of OS between patients with a high score of TIIClnc signature and a low score of TIIClnc signature in the Allen dataset. **I.** Box plot displaying the TIIClnc signature score in patients with different immunotherapy responses in the Allen dataset. **J.** Kaplan-Meier survival curve of OS between patients with a high score of TIIClnc signature and a low score of TIIClnc signature in the GSE78220 dataset. **K.** Box plot displaying the TIIClnc signature score in patients with different immunotherapy responses in the GSE78220 dataset. **L.** Kaplan-Meier survival curve of OS between patients with a high score of TIIClnc signature and a low score of TIIClnc signature in the Nathanson dataset. **M.** Box plot displaying the TIIClnc signature score in patients with different immunotherapy responses in the Nathanson dataset. **N.** Box plot displaying the TIIClnc signature score in patients with different immunotherapy responses in the GSE35640 dataset. **O.** Box plot displaying the TIIClnc signature score in patients with different immunotherapy responses in the GSE91061 dataset. **P.** Contingency table between immunotherapy responses and TIIClnc signature score groups based on TIDE algorithm in the Xiangya in-house dataset. **Q.** Contingency table between immunotherapy responses (anti-PD-1 and anti-CTLA-4) and TIIClnc signature score groups based on submap analysis in the Xiangya in-house dataset. **R.** Kaplan-Meier survival curve of OS between patients with a high score of TIIClnc signature and a low score of TIIClnc signature in the PRJNA482620 dataset.

Moreover, the TIIClnc signature was directly established in immunotherapy datasets to evaluate its predictive value. In the IMvigor dataset, urothelial carcinoma patients with high TIIClnc signature scores had a prolonged survival time (Figure 5A). As expected, urothelial carcinoma patients with high TIIClnc signature scores were more likely to respond to anti-PD-L1 immunotherapy (Figure 5B). In the Braun dataset, renal cell carcinoma patients with high TIIClnc signature scores had prolonged survival time (Figure 5D). As expected, renal cell carcinoma patients with high TIIClnc signature scores tended to respond to anti-PD-1 immunotherapy (Figure 5E). In addition, patients in the GSE179351 (colorectal adenocarcinoma and pancreatic adenocarcinoma) (Figure 5C), and GSE165252 (esophageal adenocarcinoma) (Figure 5G) datasets with high TIIClnc signature scores were also more likely to respond to immunotherapy. Notably, patients in the GSE103668 (triple-negative breast cancer) dataset with high TIIClnc signature scores were more likely to respond to targeted therapy (Figure 5F). In the Allen dataset, melanoma patients with high TIIClnc signature scores had prolonged survival time (Figure 5H). As expected, melanoma patients with high TIIClnc signature scores tended to respond to anti-CTLA-4 immunotherapy (Figure 5I). In the GSE78220 dataset, melanoma patients with high TIIClnc signature scores had prolonged survival time (Figure 5J). As expected, melanoma patients with high TIIClnc signature scores tended to respond to anti-PD-1 immunotherapy (Figure 5K). In the Nathanson dataset, melanoma patients with high TIIClnc signature scores had prolonged survival time (Figure 5L), and melanoma patients with high TIIClnc signature scores tended to respond to anti-CTLA-4 immunotherapy (Figure 5M). In addition, patients in the GSE35640 (melanoma) (Figure 5N) and GSE91061 (melanoma) (Figure 5O) datasets with high TIIClnc signature scores were also more likely to respond to immunotherapy. Based on the TIDE algorithm, a high TIIClnc signature score was significantly associated with immune checkpoint inhibitors response in the TCGA LGG (Figure S8A), Xiangya in-house (Figure 5P), CGGA LGG (Figure S8C), and GSE108474 (Figure S8E) datasets. Based on the submap analysis, a high TIIClnc signature score was related to anti-CTLA-4 and anti-PD-1 immunotherapy responses in the Xiangya in-house dataset (Figure 5Q). In contrast, a high TIIClnc signature score was mainly associated with anti-PD-1 immunotherapy response in the TCGA LGG (Figure S8B), CGGA LGG (Figure S8D), and GSE108474 (Figure S8F) datasets. Notably, in an immunotherapy dataset of glioblastoma patients, PRJNA482620, patients with high TIIClnc signature scores also had prolonged survival time (Figure 5R). The insignificant statistical analysis could be attributed to the small sample size.

**Figure 6:**
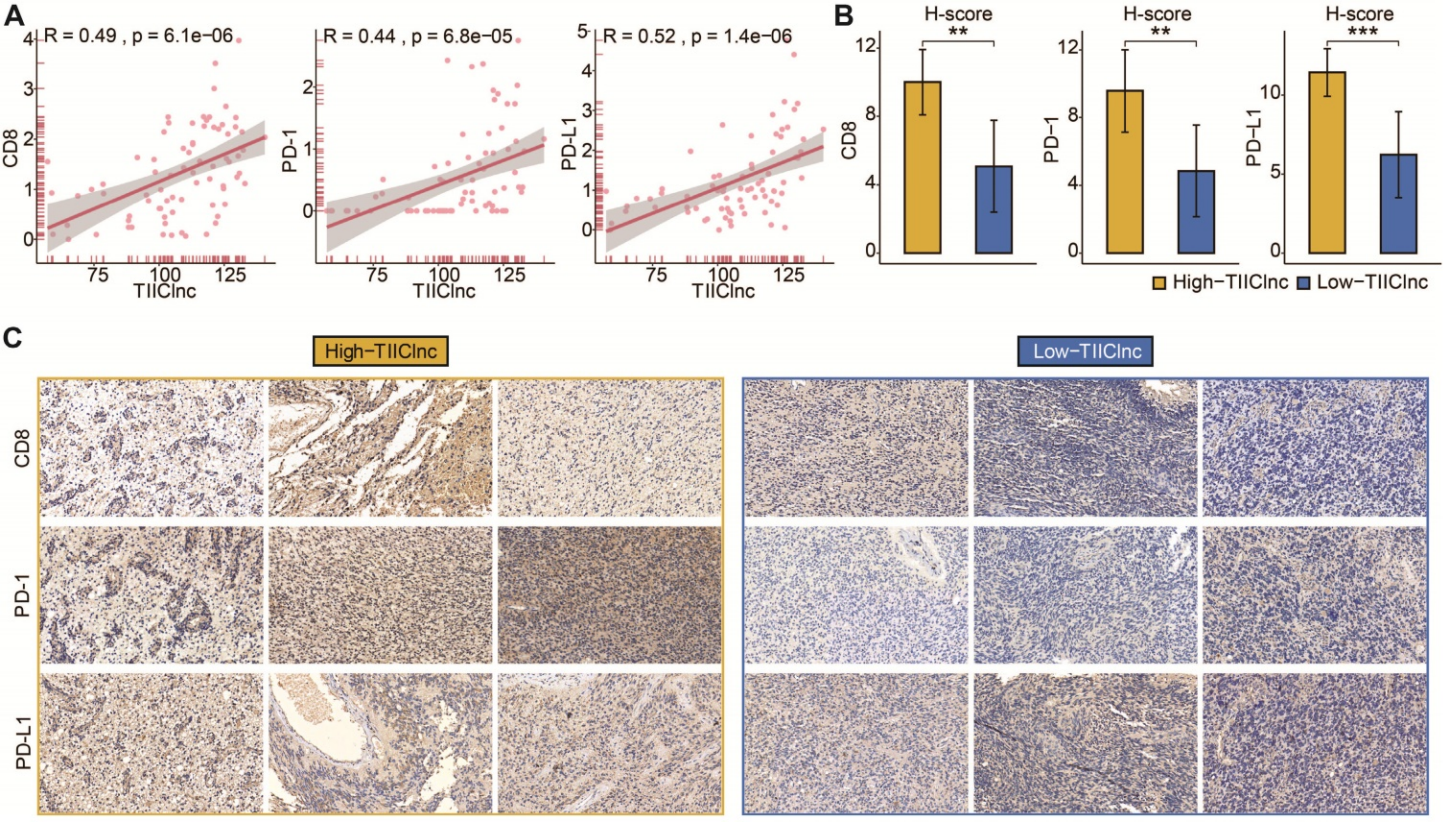
** A.** Scatter plot displaying the correlation between the TIIClnc signature score and CD8, PD-1, and PD-L1 in the Xiangya in-house dataset. **B.** Box plot displaying the H-score levels of CD8, PD-1, and PD-L1 based on IHC staining between two TIIClnc signature score groups in the Xiangya in-house dataset. The H-score was calculated by intensity score * quantity score. As for intensity scores, 0, 1, 2, and 3 were considered negative, weak, moderate, and strong, respectively. As for quantity scores, 0, 1, 2, 3, and 4 represented <10%, 10-25%, 25-50%, 50-75%, >75% proportion of stained cells, respectively. H-score ranges from 0 to 12. **C.** Representative IHC staining images of CD8, PD-1, and PD-L1 in two TIIClnc signature score groups in the Xiangya in-house dataset.

Subsequently, the predictive value of the TIIClnc signature in immunotherapy response was explored in the Xiangya in-house dataset. Scatter plots of the TIIClnc signature score and CD8, PD-1, and PD-L1 demonstrated a significant positive correlation (Figure 6A). IHC images (Figure 6B) and H-scores (Figure 6C) further proved that the protein expressions of CD8, PD-1, and PD-L1 were dramatically higher in the high TIIClnc signature score group. Our research verified that patients with a high TIIClnc signature score could benefit more from immunotherapy than those with a low TIIClnc signature score.

### Potential biological mechanisms related to the TIIClnc signature

**Figure 7:**
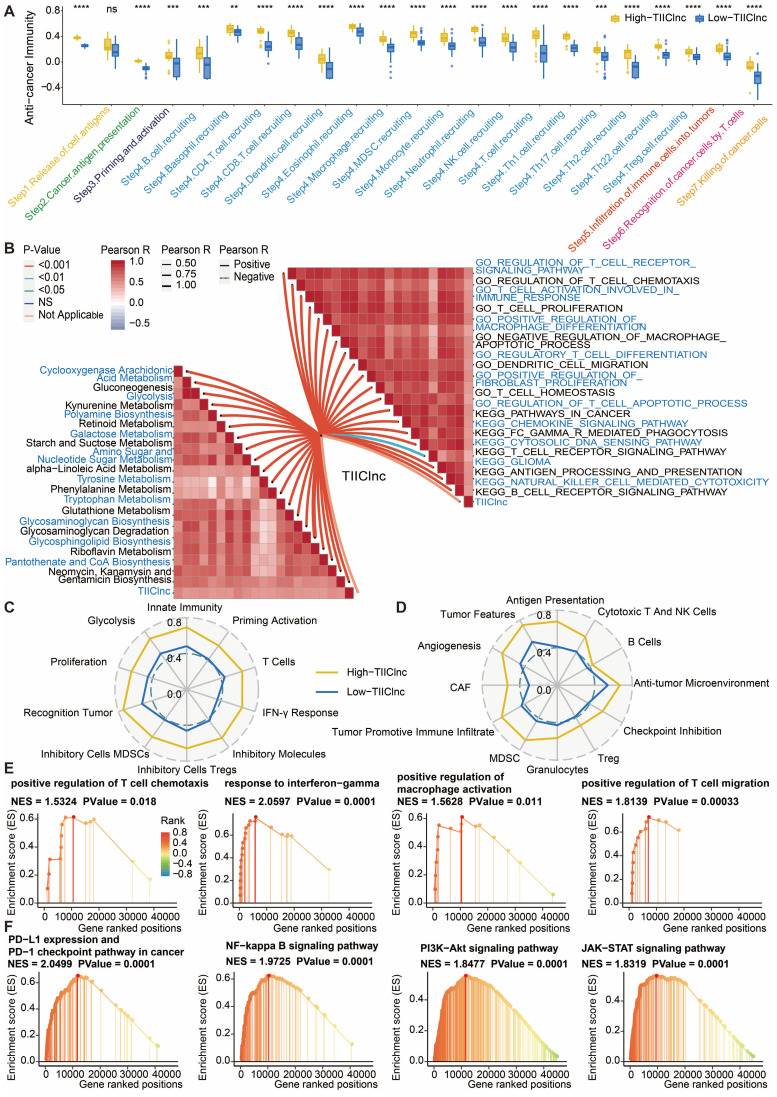
** Functional annotation of the TIIClnc signature in the Xiangya in-house dataset. A.** Box plot displaying the cancer immunity cycle differences between two TIIClnc signature score groups. **B.** Butterfly plot displaying the correlation between the TIIClnc signature score and metabolic pathways, immune-related pathways based on GSVA of GO and KEGG terms. Immunogram radar plot displaying the correlation between the TIIClnc signature score and TIME signatures developed by **C.** Kobayashi and **D.** Bagaev. **E.** GSEA of GO terms for the TIIClnc signature score. **F.** GSEA of KEGG terms for the TIIClnc signature score.

The cancer immunity cycle was calculated to explore the potential biological mechanisms related to the TIIClnc signature. Of note, all of the seven steps in the cancer immunity cycle were more activated in the high TIIClnc signature score group, including antigen release (Step 1), cancer antigen presentation (Step 2), priming and activation (Step 3), tumor immune infiltrating cells recruitment (Step 4), immune cells infiltration (Step 5), cancer cells recognition by T cells (Step 6), and cancer cells killing (Step 7) in the TCGA LGG (Figure S9A), Xiangya in-house (Figure 7A), CGGA LGG (Figure S10A), and GSE108474 (Figure S11A) datasets. The TIIClnc signature score positively correlated with the metabolic activity of metabolites, such as retinoid, glycan, galactose, or glutathione, in the TCGA LGG (Figure S9B), Xiangya in-house (Figure 7B), CGGA LGG (Figure S10B), and GSE108474 (Figure S11B) datasets. In the GSVA of GO and KEGG terms, the TIIClnc signature score positively correlated with T cell activity, macrophage activity, Treg differentiation, regulation of fibroblast proliferation, DC migration, and pathways in cancer (Figure S9B, 7B, S10B, S11B). In the analysis of TIME signatures, a high TIIClnc signature score was associated with higher levels of innate immunity, T cells, IFN-γ response, Treg, MDSC, recognition of tumor, proliferation, glycolysis developed by Kobayashi and antigen presentation, cytotoxic T and NK cells, anti-tumor microenvironment, checkpoint inhibition, Treg, granulocytes, MDSC, tumor promotive immune infiltrate, CAF, angiogenesis, tumor features developed by Bagaev in the TCGA LGG (Figure S9C and S9D), Xiangya in-house (Figure 7C and 7D), CGGA LGG (Figure S10C and S10D), and GSE108474 (Figure S11C and S11D) datasets. Additionally, in the GSEA of GO (Figure S9E, 7E, S10E, S11E) and KEGG (Figure S9F, 7F, S10F, S11F) terms, T cell chemotaxis, T cell migration, response to IFN-γ, regulation of macrophage activation, PD-L1 expression and PD-1 checkpoint pathway in cancer, PI3K-Akt pathway, and NF-kappa B pathway, JAK-STAT pathway was more enriched in the high TIIClnc signature score group.

## Discussion

Compared with glioblastoma patients, LGG patients experienced a relatively prolonged survival time of 5-10 years. However, the treatment options for LGG are still limited to chemoradiotherapy and target therapy of tyrosine kinase receptor pathway 41. The insufficient treatment options could sometimes lead to the overtreatment or undertreatment of LGG patients. Immunotherapy has become a leading star in the treatment of solid tumors. Although PD-1 has been widely detected among LGG patients, anti-PD-1 and anti-PD-L1 immunotherapies have not widely entered clinical trials of LGG patients partly due to the poor immunotherapy response and the results of several clinical trials (NCT03718767) remain to be announced 42. For this reason, establishing reliable prognostic biomarkers to stratify LGG patients who might benefit from immunotherapy is urgently needed.

This study utilized a novel computational framework to identify a robust and stable TIIClnc signature. Firstly, TSI was applied to screen out the ilncRNAs generally expressed in immune cells, indicating their fundamental roles in immune system regulation. Secondly, 101 combinations of 10 machine learning algorithms were used to identify the combination of CoxBoost and RSF as the optimal model based on the prognostic TIIClncRNAs, significantly reducing the dimensionality of variables and revealing underlying patterns, contributing to a more simplified and translational model. Therefore, the established TIIClnc signature conceivably precisely predicts LGG patients' prognosis and immune characteristics.

Among the 16 most valuable TIIClncRNAs identified for the TIIClnc signature, nine lncRNAs have been reported. C7orf13 was proved with an inverse correlation with DNA methylation in glioblastoma 43. LINC00628 contributes to lung adenocarcinoma by epigenetically interacting with the LAMA3 promoter 44. LINC01121 promotes cell proliferation, migration, and invasion of breast cancer via miR-150-5p/HMGA2 axis 45. LINC01134 dictates hepatocellular carcinoma progression by interplaying with YY1 46. SLCO4A1-AS1 could predict poor prognosis and promote proliferation and metastasis of colorectal cancer via the EGFR/MAPK axis 47. C1RL-AS1 drives the malignant phenotype of gastric cancer via the AKT/β-Catenin/c-Myc axis 48. PSMB8-AS1 leads to pancreatic cancer progression via miR-382-3p/STAT1/PD-L1 axis 49. RPARP-AS1/miR125a-5p axis promoted proliferation, migration, and invasion of colon Cancer 50. CARD8-AS1 was found to regulate the metastasis of glioma 51. The detailed reports of other 7 lncRNAs are lacking. Our research revealed that these 16 TIIClncRNAs were highly expressed in immune cells. Notably, LOC100133461 and LOC101928134 were found to inhibit the proliferation and migration ability of THP-1 cells, which was in accordance with the finding that they were hazardous markers in LGG patients. Thus, LOC100133461 and LOC101928134 were likely to facilitate tumor progression by suppressing the activity of monocytes in the TIME of LGG.

The predictive analysis demonstrated that the TIIClnc signature was a hazardous marker of OS in LGG patients. The ROC analysis further proved that the TIIClnc signature possessed high accuracy in predicting 1-year, 2-year, 3-year, 4-year, and 5-year OS of LGG patients. The stable performance of the TIIClnc signature in the TCGA LGG training dataset, Xiangya in-house dataset, and CGGA LGG, GSE108474 external validating datasets indicated the massive potential for the clinical application of the TIIClnc signature. IDH, 1p/19q, and MGMT status have long been determined as risk biomarkers for evaluating clinical strategies and outcomes of glioma patients 52, 53. Notably, the TIIClnc signature was an independent risk factor with significantly superior performance than the above three risk biomarkers and clinical factors: age, grade, and gender. In addition, 95 published signatures of various functional gene combinations were retrieved for comparison. Few of these 95 signatures have been put into clinical practice 54. Besides, many models displayed exemplary performance in the training dataset but weak performance in the validating datasets, indicating these models' poor universality and generalizability. Notably, according to the C-index assessment, the TIIClnc signature performed better than almost 95 signatures. It was conceivable that the feature gene selection and statistical prediction based on the best fit model performed by two combined machine learning algorithms ensured the stability and potential of our TIIClnc signature.

The TIME is closely associated with the prognosis of brain tumors and the efficacy of immunotherapy 55, 56. LGG patients with high TIIClnc signature score presented abundant immune cell infiltration, including T cells, Th cells, NK cells, B cells, DC, mast cells, MDSC, fibroblasts, macrophages, and Treg cells, all of which were related to anti-tumor or pro-tumor immunity in immunotherapy 57-62. Immunotherapy has demonstrated considerable benefits in cancer patients with MSI-H 63. TMB could enhance tumor immunogenicity and activate cytotoxic T cells; cancer patients with high TMB also benefit more from immunotherapy 64. In this study, LGG patients with high TIIClnc signature scores were prone to higher MSI and TMB. Meanwhile, LGG patients with high TIIClnc signature scores were also associated with higher levels of predictors for better immunotherapy responses, including CYT, GEP, TCR richness, TCR Shannon, SNV Neoantigen, IFN-γ, and IPS score. Moreover, the TIIClnc signature score was positively associated with classical immune checkpoint molecules, including IDO-1, LAG-3, PD-1, PD-L1, PD-L1, CTLA-4, BTLA, and TIGIT. For this reason, LGG patients with high TIIClnc signature scores are expected to benefit more from immunotherapy.

The TIIClnc signature was directly established in immunotherapy datasets to verify this hypothesis. The predictive analysis demonstrated that the TIIClnc signature was a favorable marker of OS in urothelial carcinoma (IMvigor dataset), renal cell carcinoma (Braun dataset), and melanoma (Allen, GSE78220, and Nathanson datasets) patients. As expected, urothelial carcinoma, renal cell carcinoma, and melanoma patients with high TIIClnc signature scores were prone to benefit more from anti-PD-1, anti-PD-L1, or anti-CTLA-4 immunotherapy, respectively. Consistently, melanoma patients in the GSE35640 and GSE91061 datasets, colorectal adenocarcinoma and pancreatic adenocarcinoma patients in the GSE179351 dataset, and esophageal adenocarcinoma patients in the GSE165252 dataset with high TIIClnc signature scores also benefit more from immunotherapy. Besides, triple-negative breast cancer patients in the GSE103668 dataset with high TIIClnc signature scores benefit more from targeted therapy. More importantly, glioblastoma patients with high TIIClnc signature scores in the PRJNA482620 dataset were related to relatively reduced survival time, which the small sample size could partly explain the insignificant results. Furthermore, in our Xiangya in-house dataset, the protein expression level of CD8, PD-1, and PD-L1 also increased in the high TIIClnc signature score group, suggesting that the TIIClnc signature could potentially predict the response rate of immunotherapy in LGG.

Although the clinical significance of the TIIClnc signature in LGG is remarkable, some limitations in this study need to be issued. First, all included datasets were from single-center retrospective studies, and future TIIClnc signature validation should be performed in prospective multicenter cohorts. Second, the in-depth molecular mechanisms of how 16 identified most valuable TIIClncRNAs could influence the TIME and immunotherapy response of LGG should be further explored. Third, more immunotherapy cohorts of glioma patients are urgently needed to validate the TIIClnc signature in predicting immunotherapy response.

In conclusion, by integrative analysis of sequencing data of purified immune cells, LGG cell lines, and bulk LGG tissues based on a wealth of machine learning algorithms, a stable and robust TIIClnc signature to stratify LGG patients and predict the outcomes of immunotherapy was developed. A TIIClnc signature is a promising tool for personalized treatment and clinical management for individual LGG patients.

## Supplementary Material

Supplementary figures.

Supplementary table 1.

Supplementary table 2.

Supplementary table 3.

Supplementary table 4.

Supplementary table 5.

Supplementary table 6.

## Abbreviations

lncRNAs: long noncoding RNAs
TIIClncRNA: tumor-infiltrating immune cell-associated lncRNA
LGG: low-grade glioma
WHO: World Health Organization
TIME: tumor immune microenvironment
ICB: immune checkpoint blockade
ACT: adoptive cell transfer
TCGA: The Cancer Genome Atlas
CGGA: Chinese Glioma Genome Atlas
GEO: Gene Expression Omnibus
CCLE: Cancer Cell Line Encyclopedia project
RMA: Robust Multi-array Average
FPKM: fragments per kilobase million
TPM: transcripts per kilobase million
TSI: tissue specificity index
RSF: random survival forest
Enet: elastic network
plsRcox: partial least squares regression for Cox
SuperPC: supervised principal components
GBM: generalized boosted regression modeling
survival-SVM: survival support vector machine
GEP: T cell-inflamed gene expression profile
CYT: cytotoxic activity
IFN-γ: interferon γ
TMB: tumor mutation burden
MSI: microsatellite instability
TCR: T cell receptor
IPS: immunophenoscore
GSVA: gene set variation analysis
GO: Gene Ontology
KEGG: Kyoto Encyclopedia of Genes and Genomes
GSEA: gene set enrichment analysis
OS: overall survival
ROC: receiver operating characteristic
BSA: bovine serum albumin
FBS: fetal bovine serum
TIMER: Tumor Immune Estimation Resource
MCPcounter: Microenvironment Cell Populations-counter
ssGSEA: single cell gene set enrichment analysis
ESTIMATE: Estimation of STromal and Immune cells in MAlignant Tumours using Expression data
