# Supplementary material for: Machine learning-based identification of tumor-infiltrating immune cell-associated lncRNAs for improving outcomes and immunotherapy responses in patients with low-grade glioma
Source: Theranostics. 2022 Aug 8;12(13):5931–48. doi: 10.7150/thno.74281 (PMC9373811; doi:10.7150/thno.74281)
Supplement: Supplementary file 1 — Supplementary figures. [file thnov12p5931s1.pdf]

Supplementary Figures

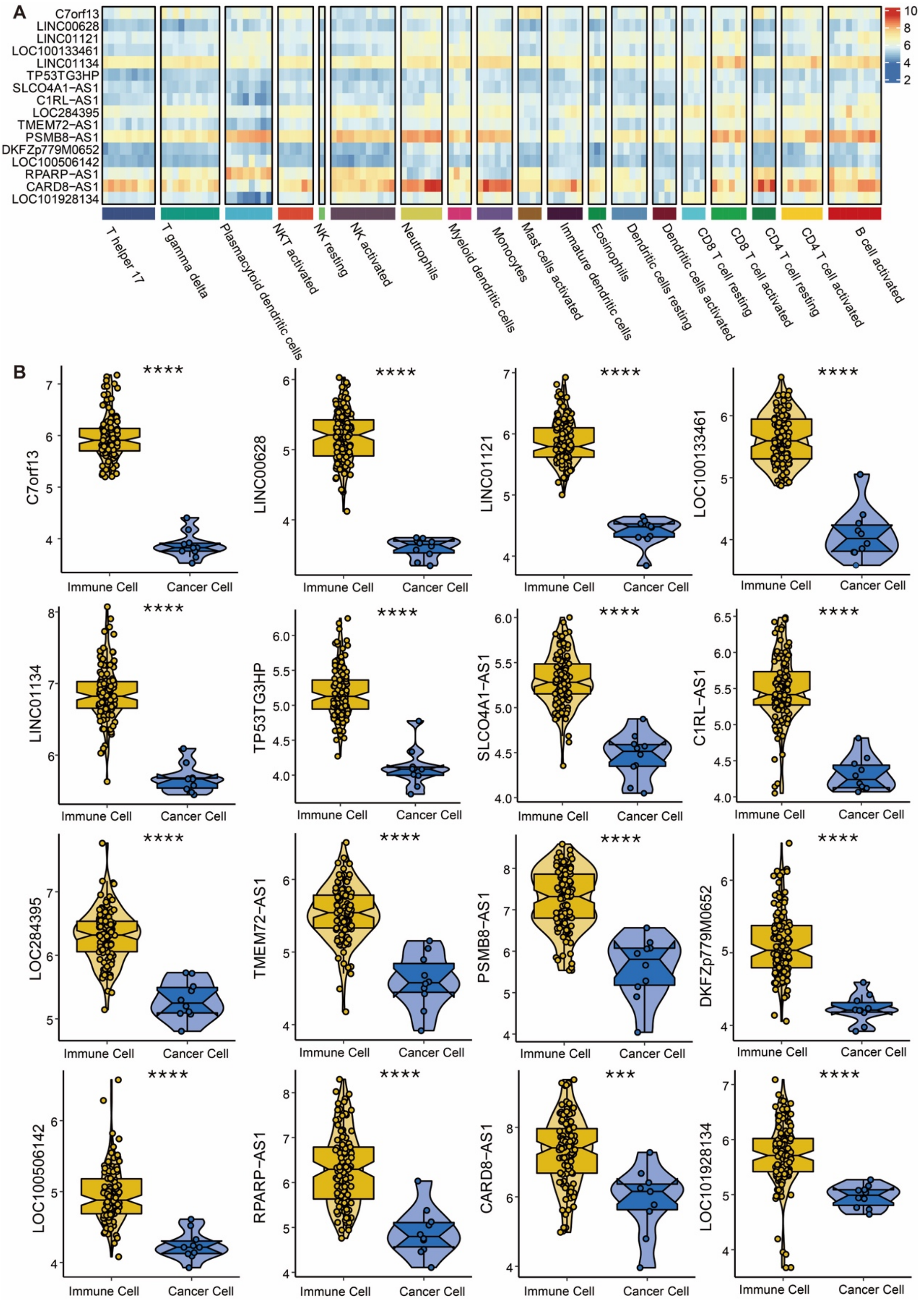

Figure S1. 16 most valuable TIIClncRNAs for TIIClnc signature. A. Heatmap

showing the expression of 16 most valuable TIIClncRNAs in 19 immune cell types. B. Box plot showing the expression of 16 most valuable TIIClncRNAs in immune cell lines and LGG cell lines.

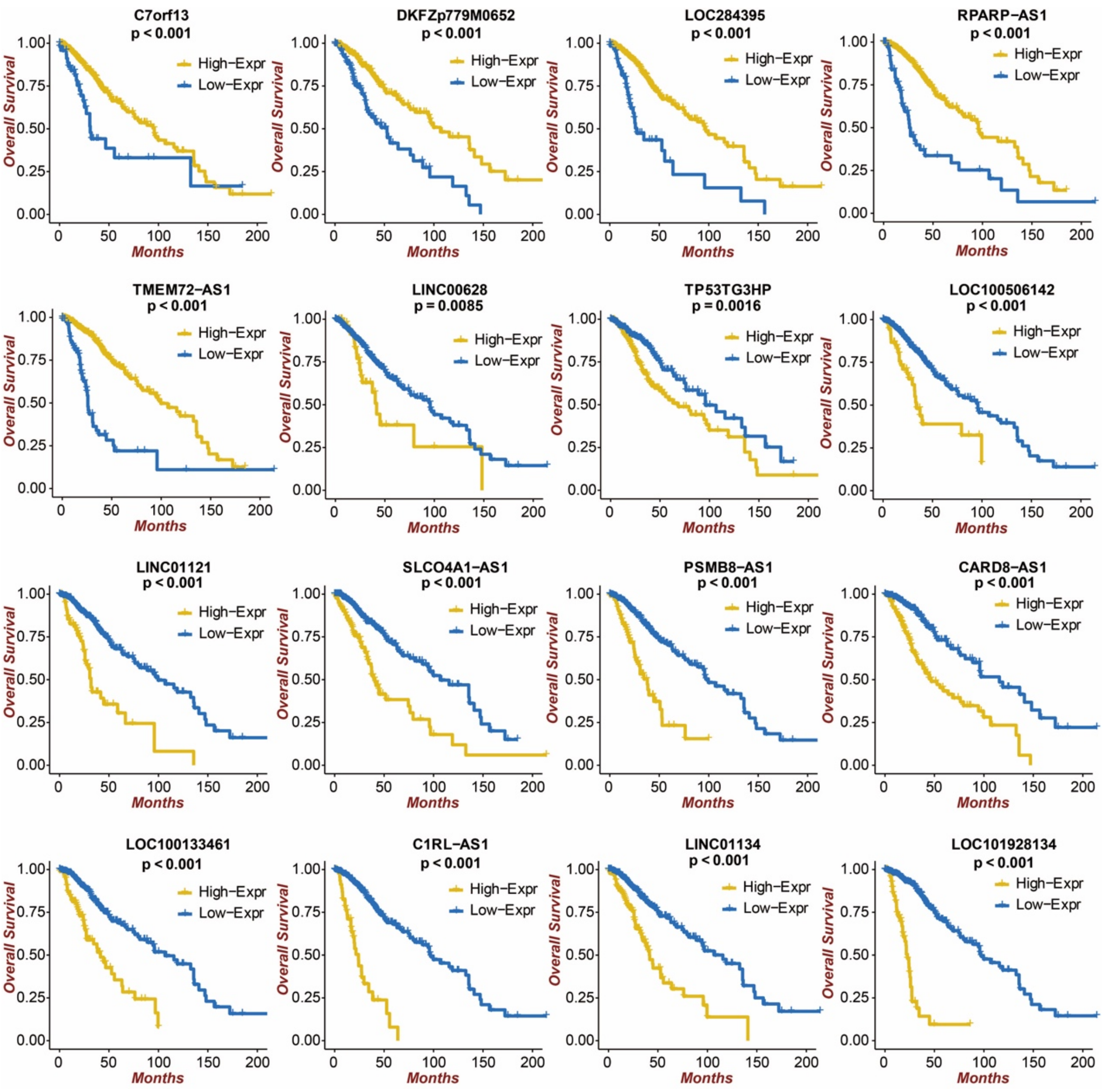

Figure S2. Kaplan-Meier survival curve of OS between patients with high or low expression of 16 most valuable TIIClncRNAs.

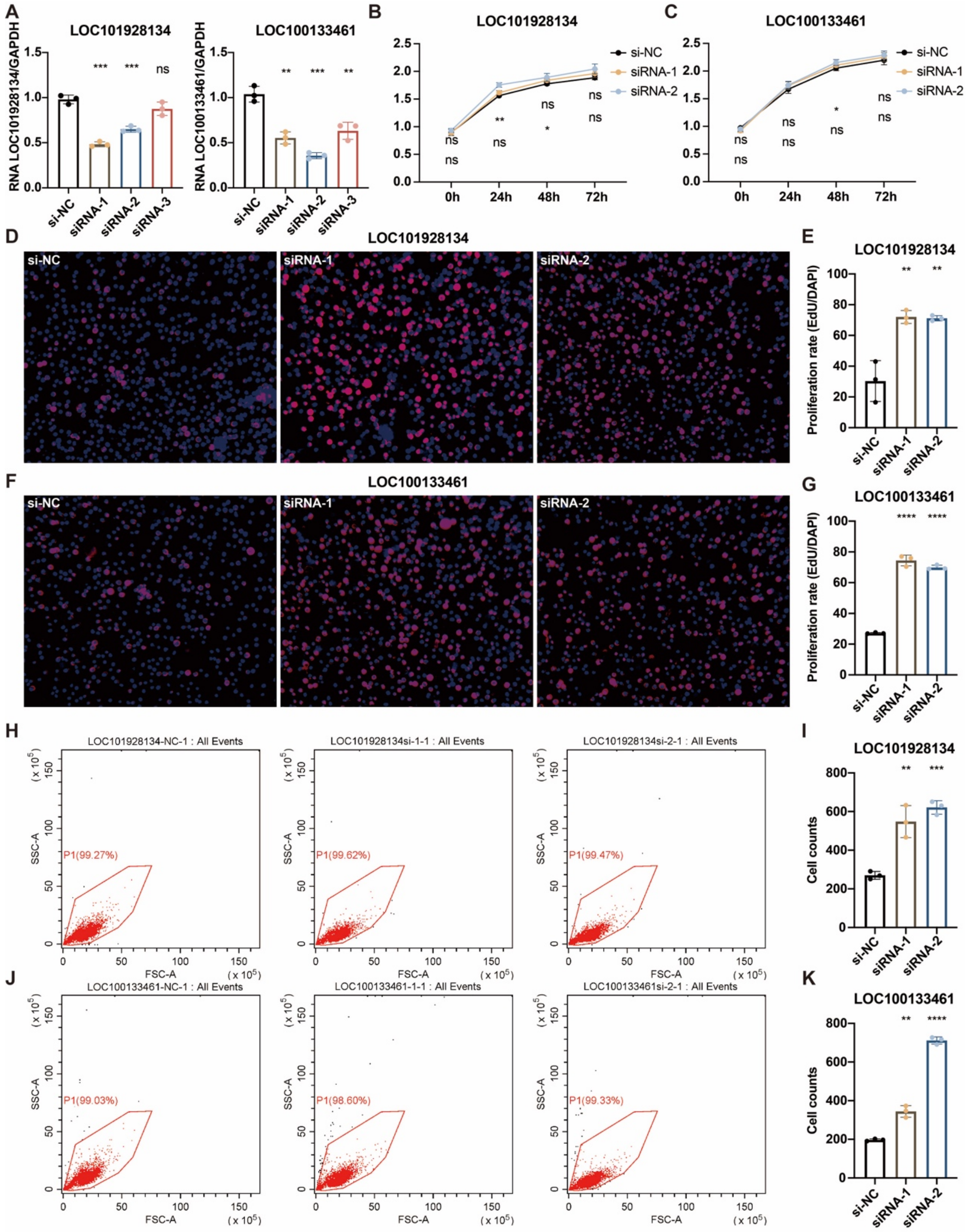

Figure S3. In vitro validation of LOC100133461 and LOC101928134. A. RNA expression of LOC100133461 and LOC101928134 in four groups of THP-1 cells transfected with siRNAs based on qPCR assay. B. CCK-8 assay of THP-1 cells transfected with siRNAs of LOC101928134. C. CCK-8 assay of THP-1 cells transfected with siRNAs of LOC100133461. D. EdU assay of THP-1 cells transfected

with siRNAs of LOC101928134. E. Statistical analysis of EdU assay. F. EdU assay of THP-1 cells transfected with siRNAs of LOC100133461. G. Statistical analysis of EdU assay. H. Flow cytometry assay of THP-1 cells transfected with siRNAs of LOC101928134. I. Statistical analysis of flow cytometry assay. J. Flow cytometry assay of THP-1 cells transfected with siRNAs of LOC100133461. K. Statistical analysis of flow cytometry assay.

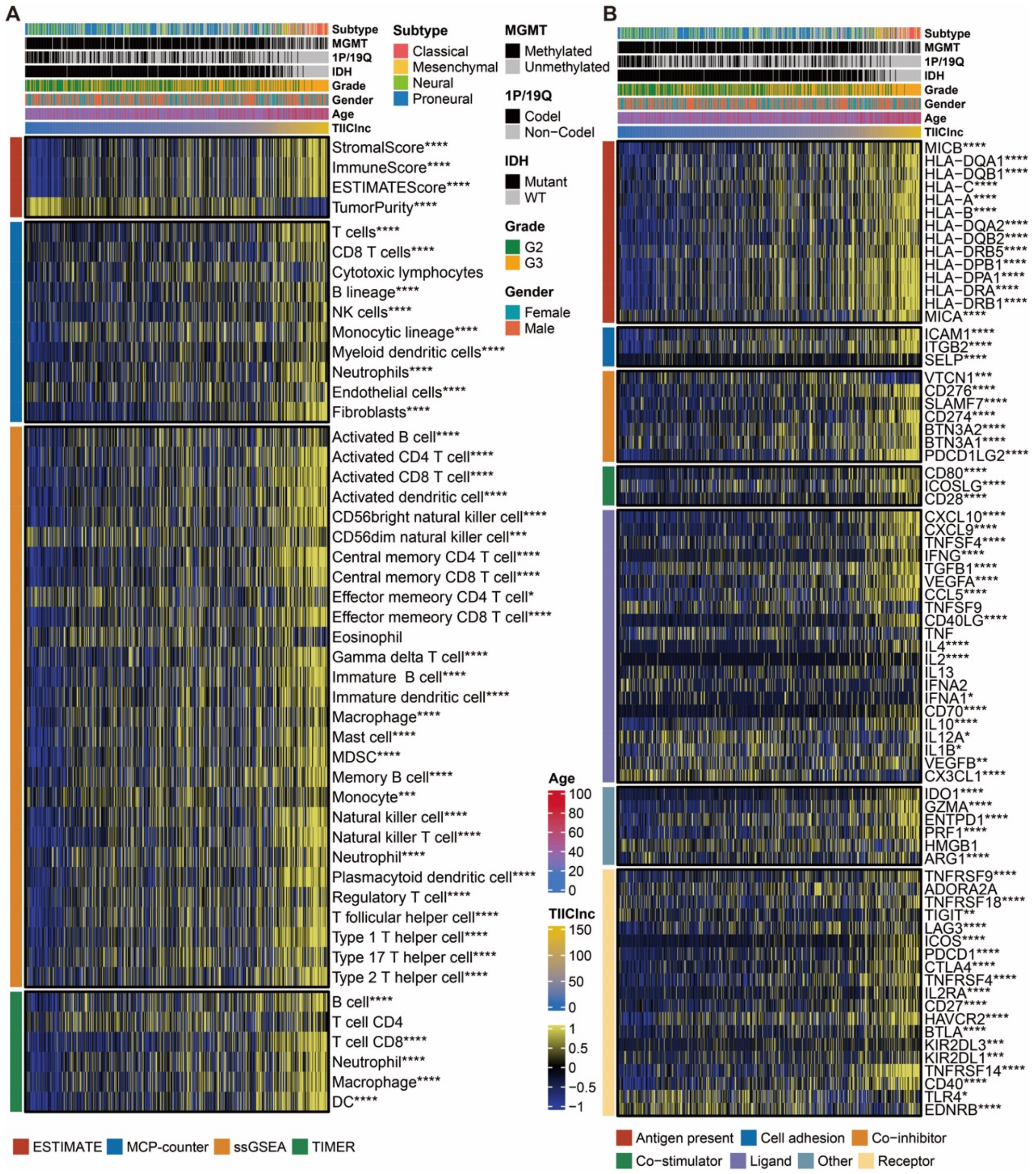

Figure S4. Immune-related characteristics of TIIClnc signature in TCGA LGG dataset.

A. Heatmap showing the correlation between TIIClnc signature and immune infiltrating cells. B. Heatmap showing the correlation between TIIClnc signature and immune modulator molecules.

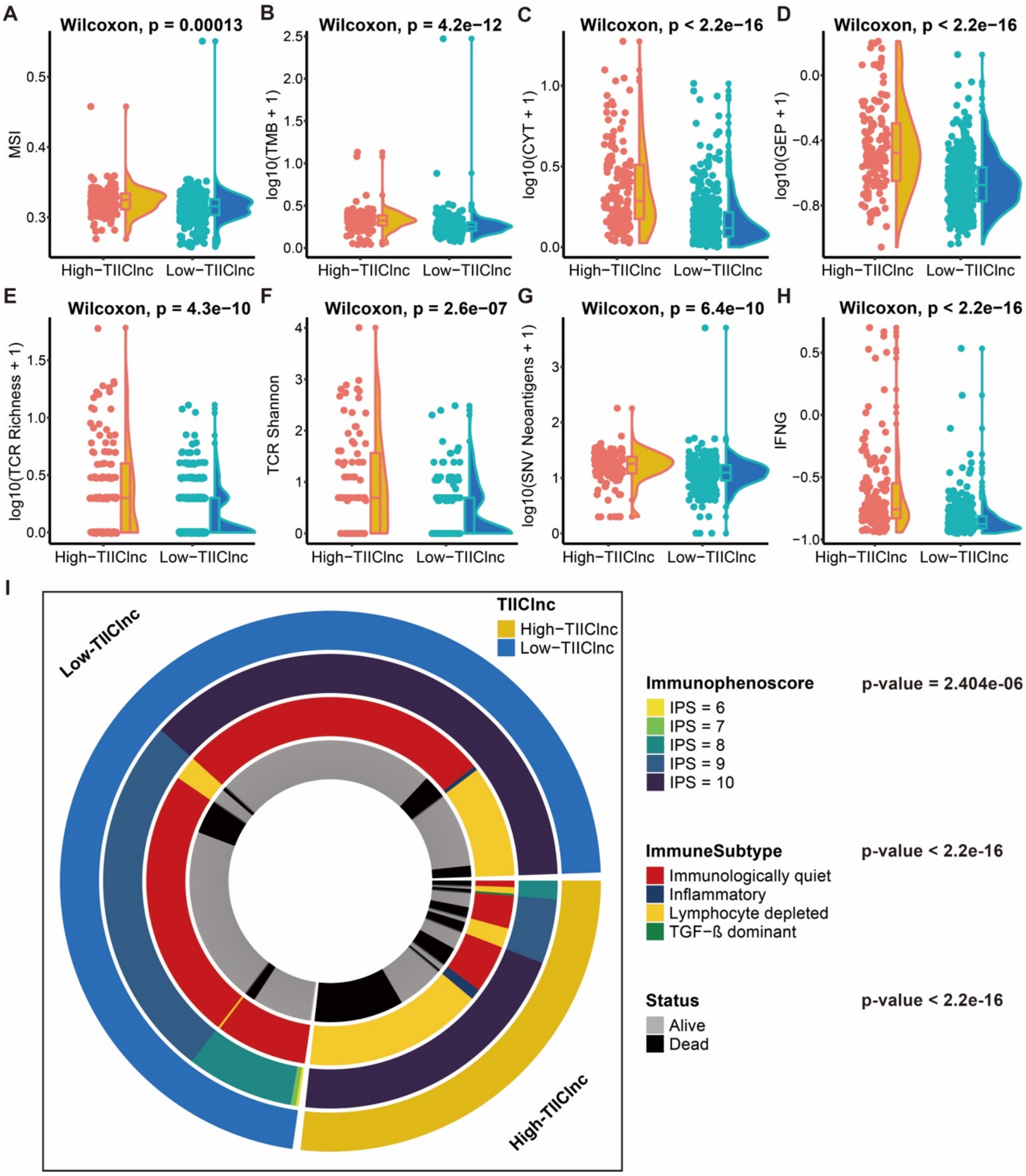

Figure S5. Relationship between TIIClnc signature and immunotherapy predictors in TCGA LGG dataset. A. Box plot showing the levels of MSI between two TIIClnc signature score groups. B. Box plot showing the levels of TMB between two TIIClnc signature score groups. C. Box plot showing the levels of CYT between two TIIClnc

signature score groups. D. Box plot showing the levels of GEP between two TIIClnc signature score groups. E. Box plot showing the levels of TCR Richness between two TIIClnc signature score groups. F. Box plot showing the levels of TCR Shannon between two TIIClnc signature score groups. G. Box plot showing the levels of SNV Neoantigen between two TIIClnc signature score groups. H. Box plot showing the levels of IFN-γ between two TIIClnc signature score groups. I. Circos plot showing the association between TIIClnc signature and six immune subtypes, IPS.

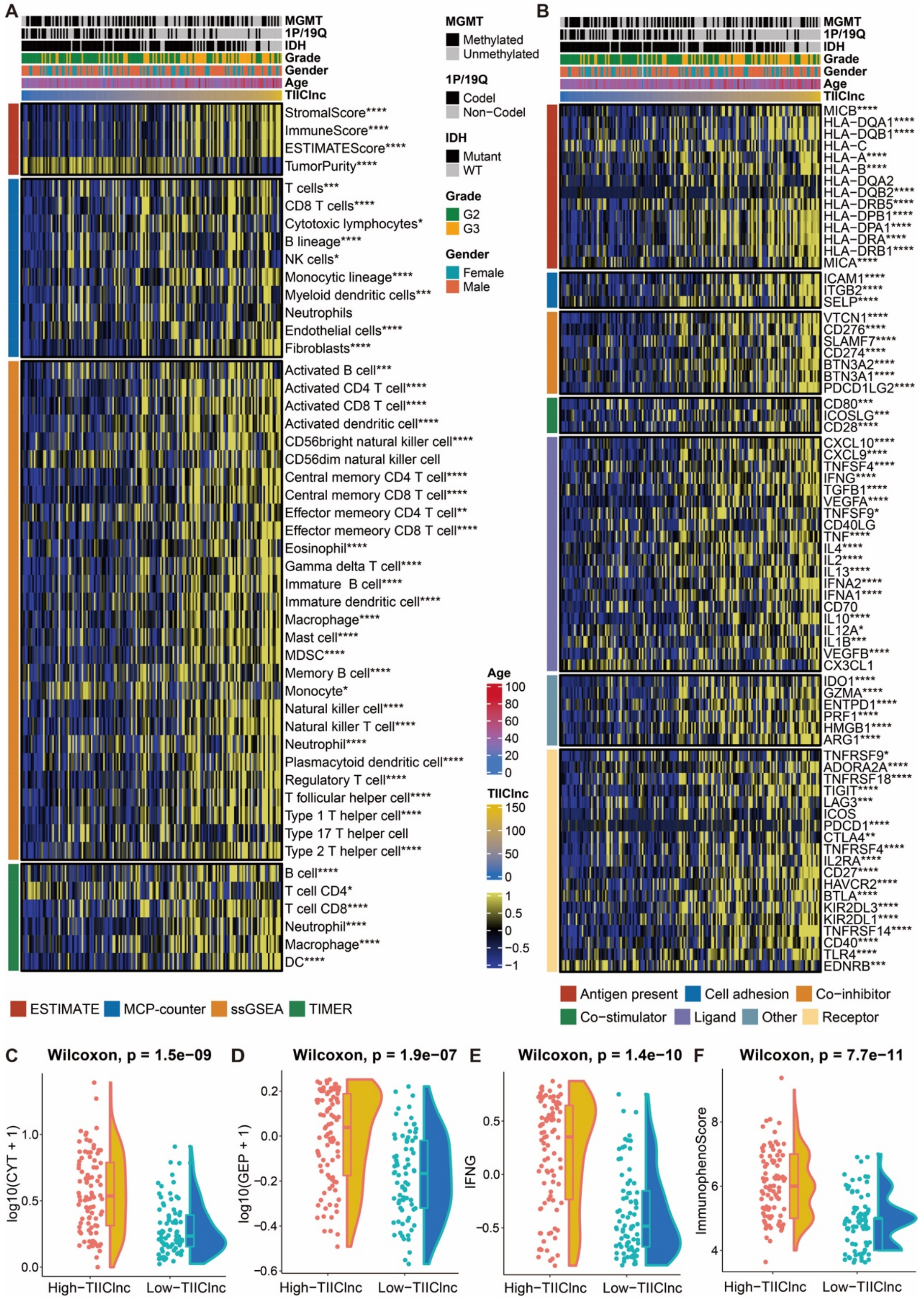

Figure S6. Immune-related characteristics of TIIClnc signature in CGGA LGG dataset.

A. Heatmap showing the correlation between TIIClnc signature and immune infiltrating cells. B. Heatmap showing the correlation between TIIClnc signature and

immune modulator molecules. C. Box plot showing the levels of CYT between two TIIClnc signature score groups. D. Box plot showing the levels of GEP between two TIIClnc signature score groups. E. Box plot showing the levels of IFN-γ between two TIIClnc signature score groups. F. Box plot showing the levels of IPS between two TIIClnc signature score groups.

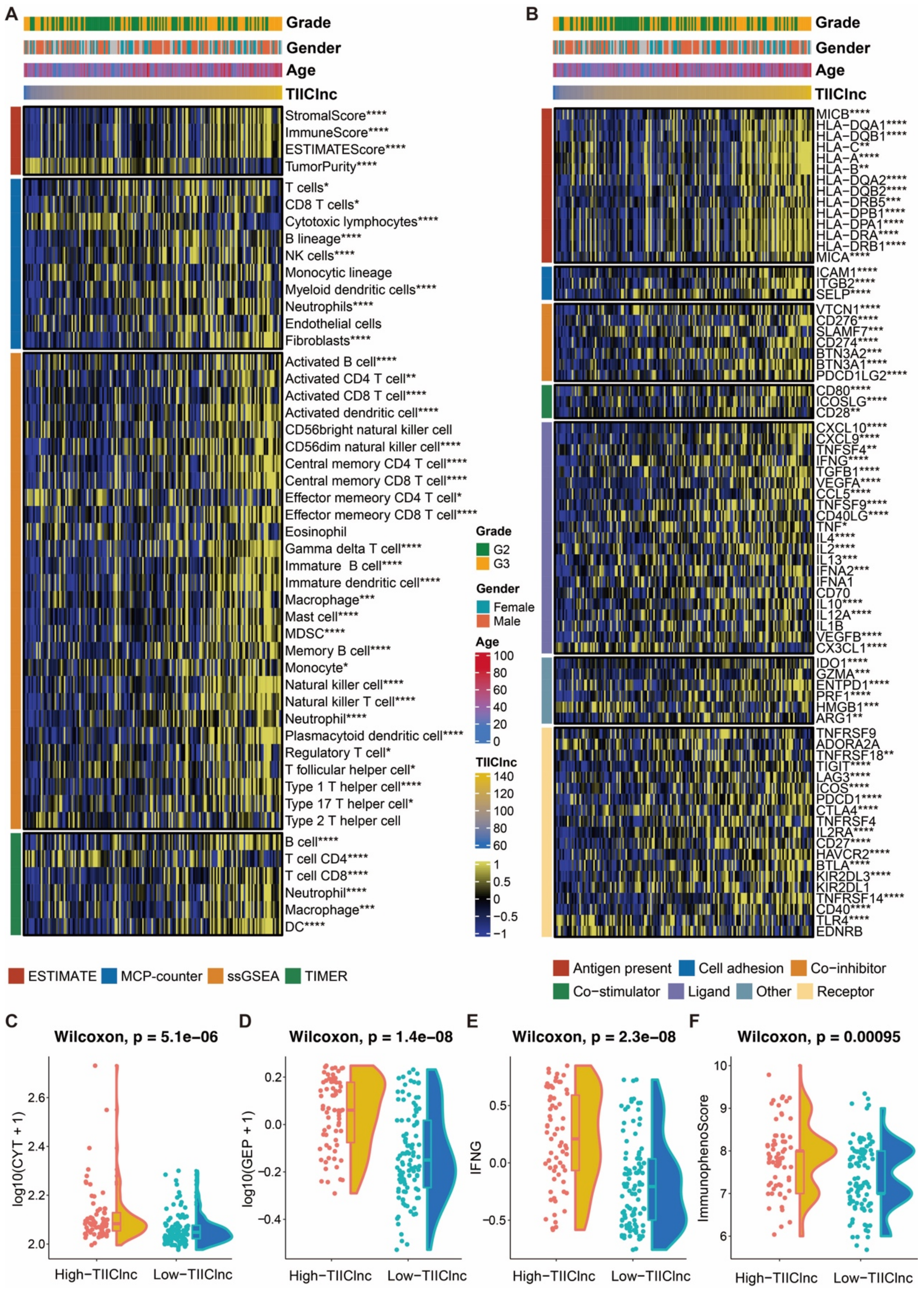

Figure S7. Immune-related characteristics of TIIClnc signature in GSE108474 dataset. A. Heatmap showing the correlation between TIIClnc signature and immune infiltrating cells. B. Heatmap showing the correlation between TIIClnc signature and

immune modulator molecules. C. Box plot showing the levels of CYT between two TIIClnc signature score groups. D. Box plot showing the levels of GEP between two TIIClnc signature score groups. E. Box plot showing the levels of IFN-γ between two TIIClnc signature score groups. F. Box plot showing the levels of IPS between two TIIClnc signature score groups.

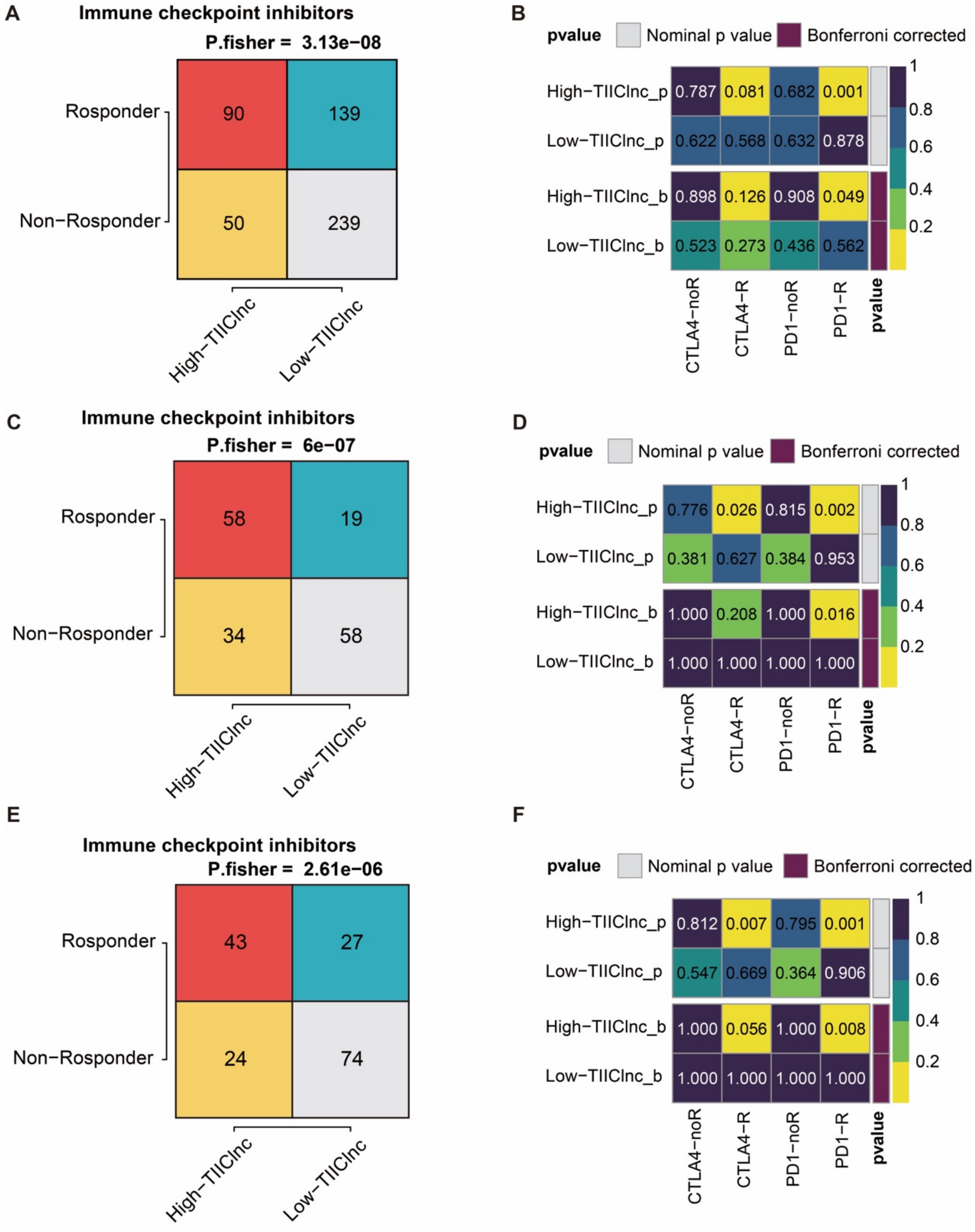

Figure S8. Contingency table between immunotherapy responses and TIIClnc signature score groups based on TIDE algorithm in A. TCGA LGG dataset, C. CGGA LGG dataset, E. GSE108474 dataset. Contingency table between immunotherapy responses (anti-PD-1 and anti-CTLA-4) and TIIClnc signature score groups based on submap analysis in B. TCGA LGG dataset, D. CGGA LGG dataset, F. GSE108474 dataset.

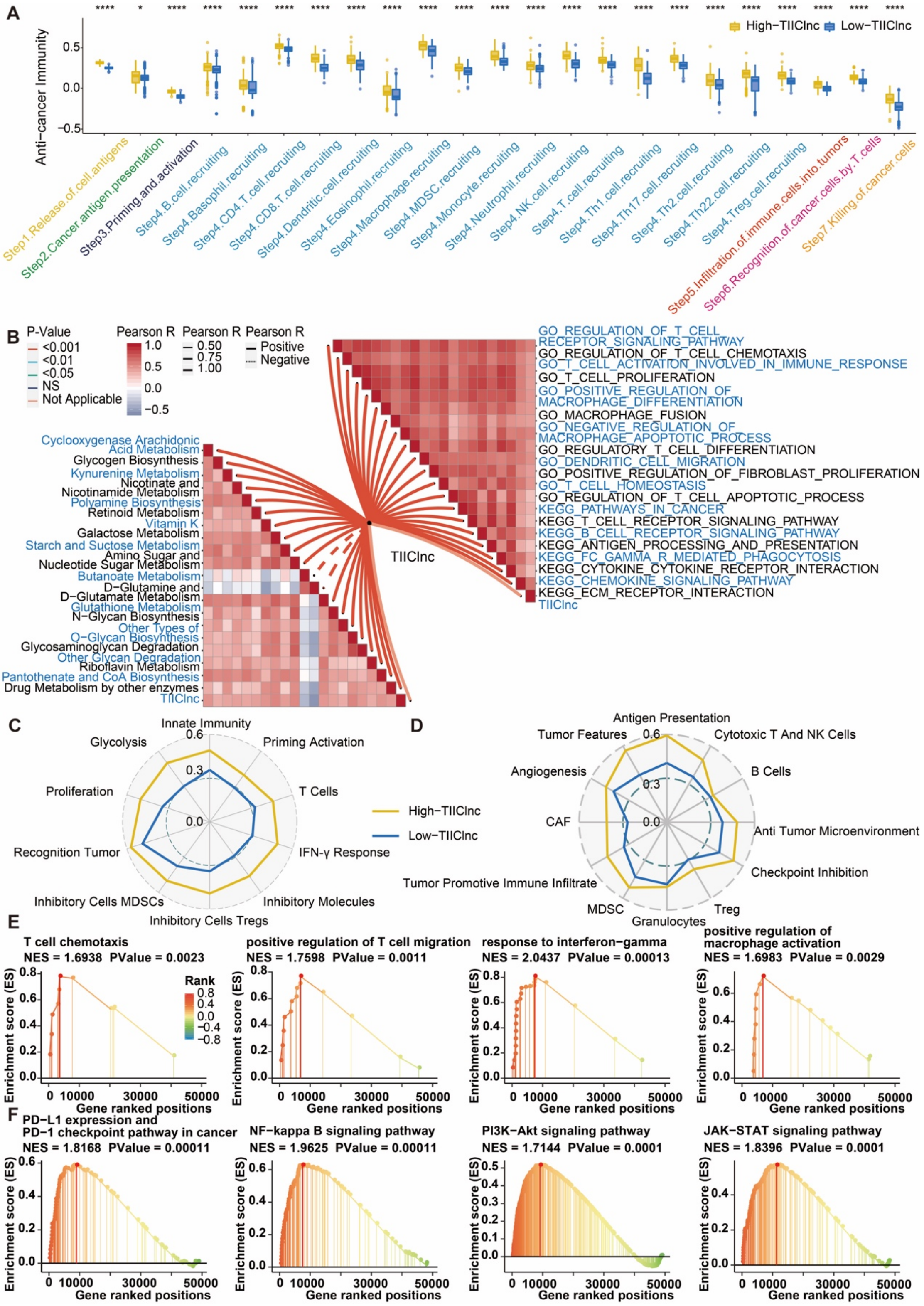

Figure S9. Functional annotation of TIIClnc signature in TCGA LGG dataset. A. Box plot showing the cancer immunity cycle differences between two TIIClnc signature score groups. B. Butterfly plot showing the correlation between TIIClnc signature

score and metabolic pathways, immune-related pathways based on GSVA of GO and KEGG terms. Immunogram radar plot showing the correlation between TIIClnc signature score and TIME signatures developed by C. Kobayashi and D. Bagaev. E. GSEA of GO terms for TIIClnc signature score. F. GSEA of KEGG terms for TIIClnc signature score.

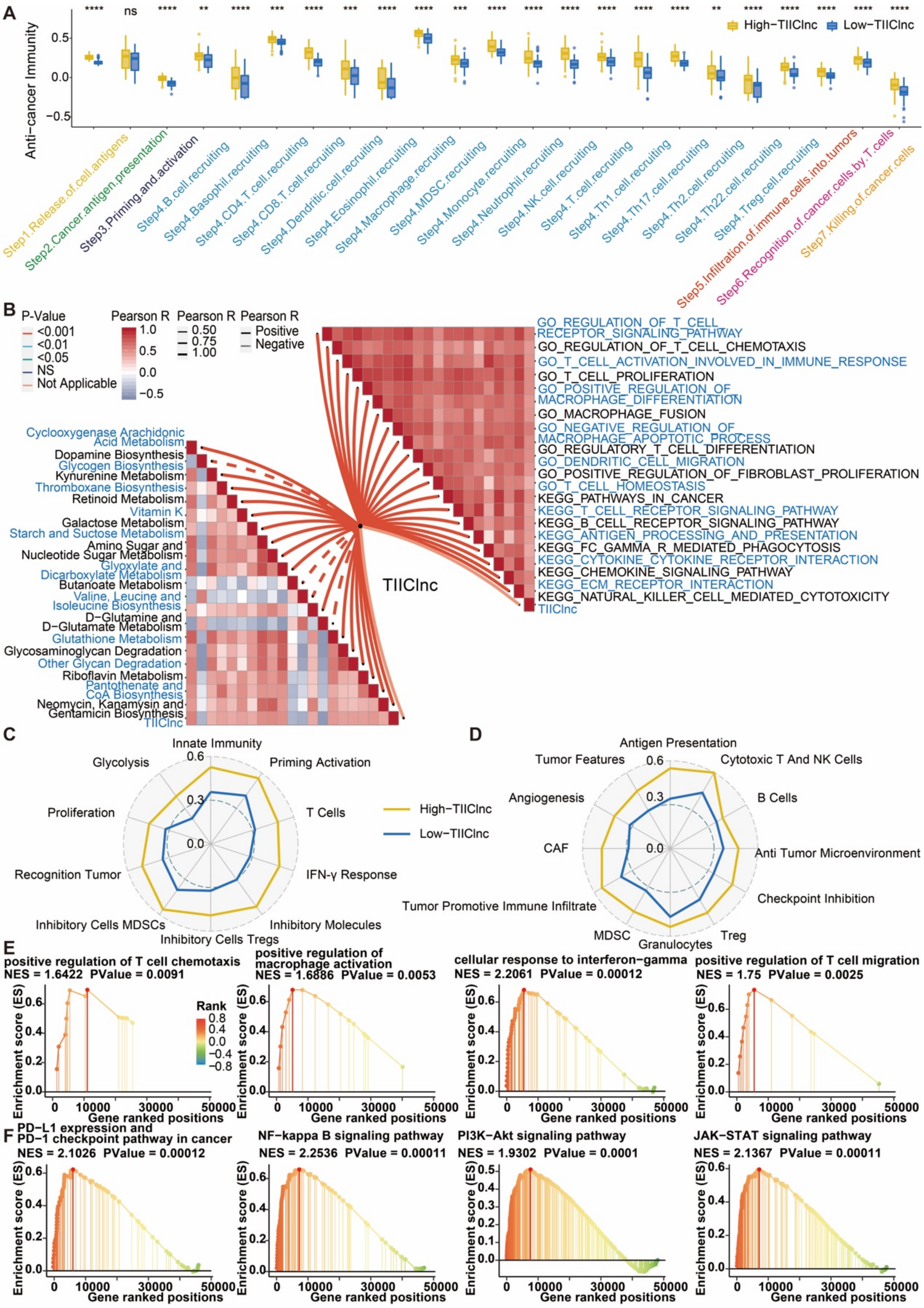

Figure S10. Functional annotation of TIIClnc signature in CGGA LGG dataset. A. Box plot showing the cancer immunity cycle differences between two TIIClnc signature score groups. B. Butterfly plot showing the correlation between TIIClnc

signature score and metabolic pathways, immune-related pathways based on GSVA of GO and KEGG terms. Immunogram radar plot showing the correlation between TIIClnc signature score and TIME signatures developed by C. Kobayashi and D. Bagaev. E. GSEA of GO terms for TIIClnc signature score. F. GSEA of KEGG terms for TIIClnc signature score.

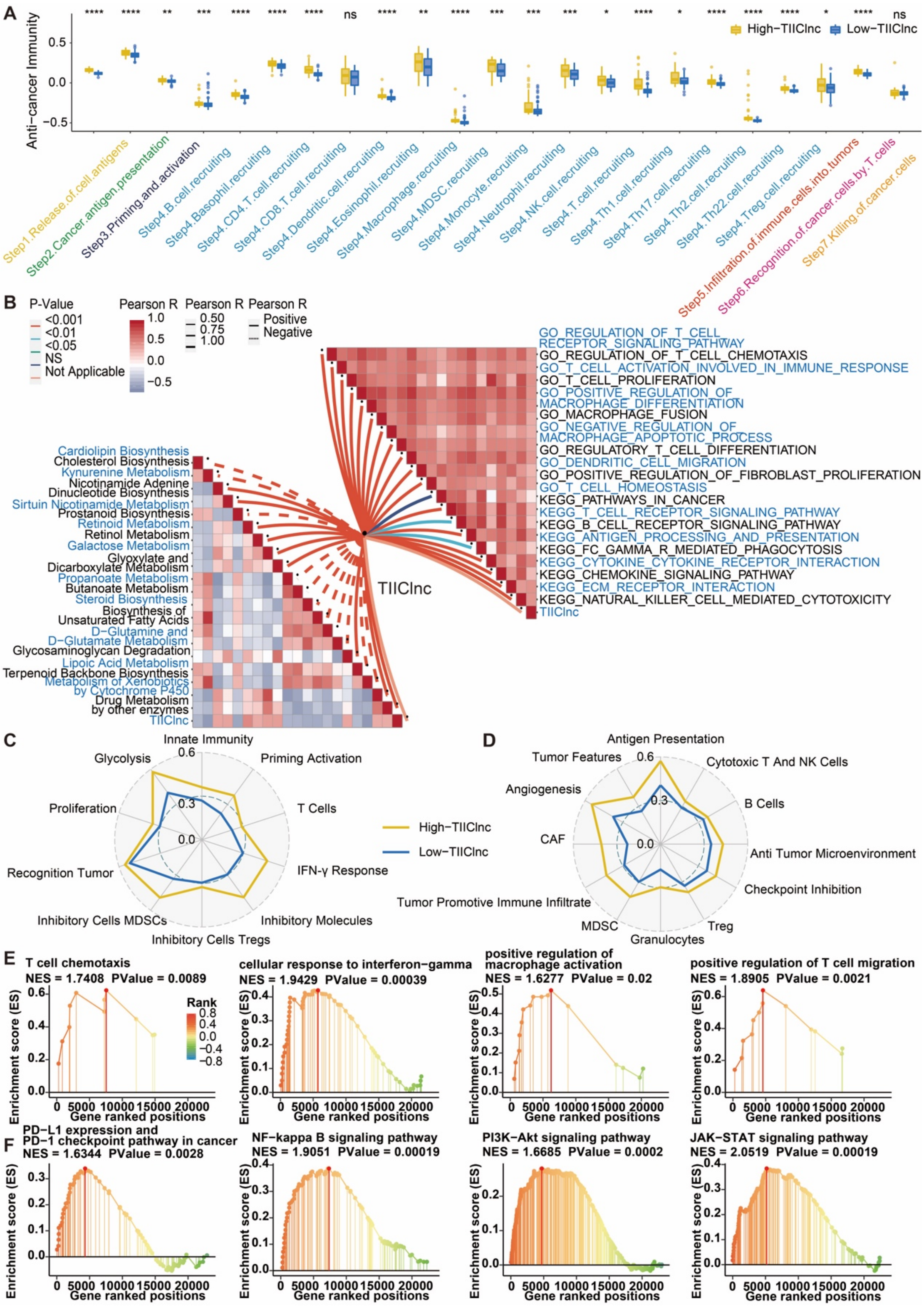

Figure S11. Functional annotation of TIIClnc signature in GSE108474 dataset. A. Box plot showing the cancer immunity cycle differences between two TIIClnc signature score groups. B. Butterfly plot showing the correlation between TIIClnc signature

score and metabolic pathways, immune-related pathways based on GSVA of GO and KEGG terms. Immunogram radar plot showing the correlation between TIIClnc signature score and TIME signatures developed by C. Kobayashi and D. Bagaev. E. GSEA of GO terms for TIIClnc signature score. F. GSEA of KEGG terms for TIIClnc signature score.
